# Association of prenatal trajectories of depressive and anxiety symptoms with neurodevelopment of children aged 0-24 months: A prospective study

**DOI:** 10.3389/fpsyt.2025.1536042

**Published:** 2025-02-18

**Authors:** Ruoqing Chen, Weiying Liu, Feng Wu, Xiaomin Ye, Xuanshu Wang, Yeqi Zheng, Weiri Tan, Ruyi Ding, Mengting Liu, Yi Gao, Hui Liang, Quanfu Zhang, Fang Fang, Yan Yu, Xu Chen, Liya Ma

**Affiliations:** ^1^ Department of Epidemiology, School of Public Health (Shenzhen), Shenzhen Campus of Sun Yat-sen University, Shenzhen, Guangdong, China; ^2^ Department of Epidemiology, School of Public Health (Shenzhen), Sun Yat-sen University, Shenzhen, Guangdong, China; ^3^ Institute of Environmental Medicine, Karolinska Institutet, Stockholm, Sweden; ^4^ Guangdong Engineering Technology Research Center of Nutrition Transformation, Sun Yat-sen University, Shenzhen, Guangdong, China; ^5^ Central Laboratory, Shenzhen Baoan Women’s and Children’s Hospital, Shenzhen, Guangdong, China; ^6^ Department of Psychology, Sun Yat-Sen University, Guangzhou, Guangdong, China; ^7^ School of Biomedical Engineering, Sun Yat-sen University, Shenzhen, Guangdong, China; ^8^ School of Medicine, Sun Yat-sen University, Shenzhen, Guangdong, China; ^9^ Department of Obstetrics, Shenzhen Baoan Women’s and Children’s Hospital, Shenzhen, Guangdong, China; ^10^ Department of Child Healthcare, Shenzhen Baoan Women’s and Children’s Hospital, Shenzhen, Guangdong, China

**Keywords:** pregnancy, depression, anxiety, child neurodevelopment, trajectory

## Abstract

**Background:**

Prenatal depression and anxiety can significantly impact a child’s neurodevelopment. However, the specific timing and patterns of these maternal psychological symptoms during pregnancy that influence the child’s neurodevelopment remain unclear. This study aimed to investigate the associations of maternal depressive and anxiety symptoms across pregnancy trimesters and their trajectories with neurodevelopmental outcomes in children aged 0 to 24 months.

**Methods:**

A total of 16,229 singletons born between January 2020 and December 2023 were included in this prospective study. Maternal depressive and anxiety symptoms in each trimester of pregnancy were assessed by Patient Health Questionnaire-9 and Generalized Anxiety Disorder-7 scale, respectively. The neurodevelopmental status of children aged 0-24 months was assessed by the Ages and Stages Questionnaire-Third Edition. Group-based trajectory model was employed to identify distinct trajectories of prenatal depressive and anxiety symptoms throughout pregnancy. Poisson regression was used to assess the associations of maternal depressive and anxiety symptoms, as well as their trajectories, with the child’s neurodevelopment.

**Results:**

A total of 1,791 (11.04%) children had neurodevelopmental delay in the communication domain, 1,127 (6.94%) in the gross motor domain, 1,750 (10.78%) in the fine motor domain, 1,137 (7.01%) in the problem-solving domain, and 1,724 (10.62%) in the personal-social domain. Maternal depressive and anxiety symptoms during pregnancy, especially in the second and third trimester, were associated with a higher risk of neurodevelopmental delay in all domains, with the most profound impact observed in the fine motor domain. Four trajectories were identified for prenatal depressive and anxiety symptoms, respectively. Compared with children whose mothers had low level of depressive or anxiety symptoms throughout pregnancy, children whose mothers experienced consistently moderate or high level of these symptoms had a higher risk of neurodevelopmental delay, while children whose mothers experienced moderate level of depressive or anxiety symptoms that decreased to low levels after the first trimester did not show a different risk of neurodevelopmental delay.

**Conclusion:**

Maternal depressive and anxiety symptoms during pregnancy have an adverse impact on the neurodevelopment in offspring. This study highlights the importance of improving the mental health of pregnant women in order to support optimal neurodevelopment for their children.

## Background

Maternal depression and anxiety during pregnancy have become a major global public health concern, impacting the well-being of both mothers and their offspring (1, 2). Prenatal depression and anxiety have been associated with adverse pregnancy outcomes, including gestational hypertensive disorders, gestational diabetes, and postpartum depression, as well as adverse birth outcomes, such as preterm birth, low birth weight, lower Apgar scores and longer neonatal intensive care stays, etc (3–9). Beyond these immediate consequences, prenatal mental health can also have a long-term impact on the children’s developmental outcomes (10–13).

Primary neurulation commences around 18 days of gestation, with the neural tube completing its closure by days 29 to 30 (14). Following this, the fetal brain undergoes rapid expansion, engaging in critical processes such as neural formation, cell proliferation, and synapse development, which lay the foundation for neurodevelopment postnatally (15). Neurogenesis is predominantly completed early in pregnancy, with neuronal migration reaching its peak by 15 weeks of gestation (16). The mid-pregnancy period is characterized by intense synapse formation, dendrite growth, axonal pathfinding, and hippocampal neurogenesis, essential for sensory processing, communication, emotions and memory (16, 17). In late pregnancy, thalamocortical connections strengthen, long-range fibers such as the corpus callosum extend, and cortical surface area increases, supporting sensory-motor integration and social-emotional development. Meanwhile, functional connectivity enhances, with key brain hubs establishing long-distance connections among sensory, motor, and higher-order regions. Collectively, these changes lay the groundwork for neural circuits essential for cognitive, emotional, and social functions following birth (16, 18).

Prenatal maternal factors play a crucial role in influencing early neurodevelopment. Maternal conditions, including overweight or obesity, hypertensive disorders, diabetes, and mental health issues such as depression or anxiety during pregnancy, have been linked to an increased risk of neurodevelopmental problems in children (19). In particular, maternal depression and anxiety may disrupt the critical brain development period by interfering with fetal programming, potentially resulting in adverse effects on children’s neurodevelopment (20–24). Several large cohort studies from the United Kingdom, Netherlands and Canada found that maternal depression and anxiety during pregnancy adversely affect early child development, leading to general psychopathology, internalizing problems, and poorer social and emotional outcomes (25, 26). A prospective cohort study conducted in England found that prenatal depression was associated with poorer adaptive behavior in infants (20). A birth cohort study in Shanghai, China found that prenatal maternal depression and anxiety adversely affected infant social-emotional and communication development at 24 months of age and fine motor development in girls at 6 and 12 months of age (21). Similarly, another cohort study in Shanghai found that maternal anxiety and depressive symptoms in late pregnancy were associated with poorer neurodevelopment in children at 2, 6, 12, and 24 months of age, especially in personal-social domain (27). Delays in these domains before 24 months of age have been found to predict developmental delays at 48 months and cognitive delay at 6-8 years of age (28, 29).

However, much of the existing evidence was derived from studies that focused on specific trimesters rather than encompassing the entire pregnancy (21, 27, 30, 31). While trimester-specific analyses provide valuable insights into the effects of maternal depression and anxiety at distinct gestational periods, they fail to capture the dynamic nature of maternal mental health throughout pregnancy. Maternal depressive or anxiety symptoms can fluctuate over time; some mothers may experience persistent symptoms, while others may recover or develop these symptoms later in their pregnancy (23, 32). Employing trajectory analysis allows us to identify different patterns of maternal psychological status across pregnancy and examine whether persistent or changing exposure to maternal depressive and anxiety symptoms differentially impacts children’s neurodevelopmental outcomes. This is important for recognizing maternal prenatal depressive and anxiety as modifiable factors that can be targeted to improve children’s neurodevelopment outcomes.

In this prospective study, we aimed to investigate the impact of maternal depressive or anxiety symptoms during the three trimesters of pregnancy, as well as their trajectories, on the neurodevelopmental outcomes of children aged 0 to 24 months.

## Methods

### Study design and data sources

This study was performed at Shenzhen Baoan Women’s and Children’s Hospital, China. Data utilized in this study, including sociodemographic information, medical records from pregnancy to delivery for mothers, and routine health check-ups for children, were obtained from the hospital information system, a routinely updated electronic healthcare database that contains information from the entire hospital. All mothers and children were assigned a unique healthcare identification number, which facilitated the matching of mother-child dyads and enabled information integration across different databases within the hospital information system.

### Study population

A total of 68,859 singletons were born at Shenzhen Baoan Women’s and Children’s Hospital between January 2020 and December 2023. Among these children, 46,820 children whose mothers underwent at least one prenatal assessment of depression and anxiety were included. All children were invited to undergo health check-ups, including neurodevelopmental assessments, in the same hospital.

### Exposure

The exposure of interest was defined as the presence of prenatal depressive or anxiety symptoms. Each pregnant woman was requested to undergo one psychological assessment at each trimester, specifically at 0-13 weeks, 14-27 weeks, and after 28 weeks of gestation. Depressive symptoms were assessed using the self-administered Patient Health Questionnaire-9 (PHQ-9), while anxiety symptoms were assessed using the Generalized Anxiety Disorder-7 (GAD-7) scale.

The PHQ-9 is a questionnaire recommended by the National Health Commission of China in the first national guideline for the prevention and treatment of perinatal depression (33). It was widely used and validated in the Chinese population, including pregnant women, for assessing the presence and severity of depressive symptoms (34–36). The PHQ-9 consists of nine questions that assess the frequency of depressive symptoms over the past two weeks. Each answer is scored from zero to three, and the total score is divided into five categories: 1) no or minimal depression (0-4), 2) mild depression (5-9), 3) moderate depression (10-14), 4) moderately severe depression (15-19), and 5) severe depression (20-27) (37). In this study, a score above 4 was regarded as the presence of depressive symptoms (38).

The GAD-7 is a questionnaire designed to assess the presence and severity of anxiety symptoms (39–41). It has been validated for use in pregnant Chinese women (42). The GAD-7 consists of seven items, with each answer scored on a scale from zero to three. The total score is classified into four categories:1) no or minimal anxiety (0–4), 2) mild anxiety (5–9), 3) moderate anxiety (10–14), and 4) severe anxiety (15 or above) (43). In this study, a score above 4 represented the presence of anxiety symptoms (44).

### Outcome

The outcome of interest was neurodevelopmental delay assessed by the Chinese version of the Ages & Stages Questionnaire-Third Edition (ASQ-3) during the health check-ups. The ASQ-3 has demonstrated high reliability and validity within the Chinese population (45). This 30-item parent-reported questionnaire evaluates the neurodevelopment of children aged 1-66 months across five domains: communication, gross motor, fine motor, problem-solving, and personal-social. Each domain is assessed with six questions. Each question is scored as 0, 5, or 10 points, and a total score is assigned to each domain. The scores are compared with the cut-off values of each domain to determine a child’s neurodevelopmental status. Scores less than or equal to the mean minus standard deviation (SD) but greater than the mean minus 2SD are considered at risk for potential developmental delay. Scores less than the mean minus 2SD are considered delayed development (46). In this study, neurodevelopmental delay was defined as a score less than or equal to the mean minus SD in a specific domain. Children who did not have any ASQ-3 assessments between 0 and 24 months of age (N=30,591) were excluded from the study, resulting in 16,229 children in the final analysis (11,516, 3,082, and 1,631 children assessed at the age of 0-6, 7-12, 13-24 months, respectively).

### Confounders

Potential confounders were determined based on previous literature, which identified variables associated with both maternal depression or anxiety during pregnancy and child neurodevelopment. These confounders included maternal age at delivery (47), educational level (48, 49), parity (48, 49), mode of conception (50, 51), pre-pregnancy body mass index (BMI) (52, 53), diabetic diseases (9, 54), hypertensive diseases (55), vaginal bleeding during early pregnancy (56), as well as sex (57) and calendar year of birth of the child. Information on these confounders was obtained from the hospital information system. Pre-pregnancy BMI was calculated by the pre-pregnancy weight (in kilograms) divided by the square of the measured height (in meters) and further categorized into four groups according to the recommendation by the Working Group on Obesity in China: <18.5 kg/m^2^, 18.5-23.9 kg/m^2^, 24.0-27.9 kg/m^2^, and ≥28 kg/m^2^ (58). Diabetic diseases included pregestational and gestational diabetes mellitus. Hypertensive diseases consisted of pregestational hypertension, gestational hypertension, preeclampsia, and eclampsia.

### Statistical analysis

We described the characteristics of the study population, including maternal factors such as age at delivery, educational level, parity, mode of conception, pre-pregnancy BMI, history of diabetes, hypertensive diseases, and vaginal bleeding during early pregnancy, as well as children’s characteristics, including sex, gestational age at birth, birth weight, and calendar year of birth. We then calculated the cumulative incidence of neurodevelopmental delay across the five domains and compared the distribution of these characteristics between children with and without developmental delays in each domain. Continuous variables were presented by mean and standard deviation or median and interquartile range, while categorical variables were presented as frequency and percentage.

To explore the trajectories of maternal depressive or anxiety symptoms throughout pregnancy, we employed a group-based trajectory model using the TRAJ package in STATA. This statistical model allows for the identification of distinct subgroups within a population that follow different trajectories over time (59). Mothers who underwent three prenatal psychological assessments were included in this analysis and classified into distinct trajectory groups based on their depression and anxiety scores across the three trimesters of pregnancy. Given the predominance of zero scores for depressive or anxiety symptoms, a zero-inflated distribution model was applied within the TRAJ package. To determine the best-fitting model, we initially tested models with three to six trajectories, selecting the optimal number of trajectories based on the highest Bayesian Information Criteria (BIC) values and ensuring that each trajectory group accounted for at least 5% of the population. This process identified a four-trajectory solution as the optimal model for both depressive and anxiety symptoms (**Supplementary Table S1**). Subsequently, we refined the models by testing combinations of linear and quadratic terms to determine the best trajectory shapes. The final model was selected based on the highest BIC values, average posterior probabilities above 0.7, and odds of correct classification exceeding 5 (59). Eventually, a four-trajectory model with quadratic terms was determined for both depressive and anxiety symptoms (**Supplementary Table S2**).

Poisson regression was conducted to estimate the risk ratio (RR) and 95% confidence intervals (CI) of neurodevelopmental delay in children of mothers with either prenatal depressive or anxiety symptoms, as well as mothers in different trajectories of prenatal depressive or anxiety symptoms. In the multivariable regression analysis, RRs were adjusted for the confounders described above. To address potential bias from missing values of confounders (1.42%, 1.45%, 1.47%, and 11.44% for mode of conception, maternal educational level, pre-pregnancy BMI, and vaginal bleeding during early pregnancy, respectively), we further examined the associations using multiple imputations by chained equations. Ten imputations with 50 iterations each were performed.

To assess whether the associations were consistent and robust across different age groups, we performed stratification analyses by age of the child at the time of the ASQ-3 assessment. To assess whether the associations were modified by sex of the child, we performed stratification analyses according to sex. Both analyses were tested for interaction effects and adjusted for the same confounding factors as those used in the primary analyses.

STATA version 17 (StataCorp, College Station, TX, USA) and R version 4.4.1 (R Foundation for Statistical Computing, Vienna, Austria) were used for statistical analysis and plotting.

## Results

Among 16,229 children, 1,791 (11.04%) had a neurodevelopmental delay in the communication domain, 1,127 (6.94%) in the gross motor domain, 1,750 (10.78%) in the fine motor domain, 1,137 (7.01%) in the problem-solving domain, and 1,724 (10.62%) in the personal-social domain. Children born to mothers who received assisted reproductive technology or had diabetic diseases, delivered at 33 gestational weeks or earlier, with a birth weight below 2500 grams, or those who were male, were more likely to have a higher cumulative incidence of neurodevelopmental delay across all five domains (**Supplementary Table S3**).

A total of 9,728 mothers underwent assessment for depression in the first trimester, 11,158 in the second trimester, and 12,130 in the third trimester. Overall, 3,819 (39.26%) showed depressive symptoms in the first trimester, 1,652 (14.81%) in the second trimester, and 1,948 (16.06%) in the third trimester. Maternal depressive symptoms during any trimester of pregnancy were associated with an increased risk of neurodevelopmental delays in five domains for children, except for a lack of association between depressive symptoms in the first trimester and delays in the problem-solving domain, after confounding adjustment. The strongest associations were found for the fine motor domain (second trimester: RR: 1.41, 95% CI: 1.22, 1.62; third trimester: RR: 1.41, 95% CI: 1.23, 1.60), followed by gross motor (second trimester: RR: 1.39, 95% CI: 1.15, 1.67), personal-social (third trimester: RR: 1.36, 95% CI: 1.18, 1.55), problem-solving (third trimester: RR: 1.31, 95% CI: 1.10, 1.56), and communication domains (first trimester: RR: 1.19, 95% CI: 1.06, 1.34; third trimester: RR: 1.19, 95% CI: 1.03, 1.36) (**Table 1**). The associations became more pronounced in the multiple imputation analysis (**Supplementary Table S4**).

**Table 1:** Association between prenatal depressive symptoms across the three trimesters of pregnancy and risk of neurodevelopmental delay among children.

Prenatal depressive symptoms	Total (N)	Communication		Gross motor		Fine motor		Problem-solving		Personal-social	
		No. of cases (Cumulative incidence^1^)	RR (95% CI)^2^	No. of cases (Cumulative incidence^1^)	RR (95% CI)^2^	No. of cases (Cumulative incidence^1^)	RR (95% CI)^2^	No. of cases (Cumulative incidence^1^)	RR (95% CI)^2^	No. of cases (Cumulative incidence^1^)	RR (95% CI)^2^
First trimester (N=9,728)											
No	5,909	620 (104.92)	Ref.	422 (71.42)	Ref.	603 (102.05)	Ref.	428 (72.43)	Ref.	628 (106.28)	Ref.
Yes	3,819	477 (124.90)	1.19 (1.06, 1.34)	320 (83.79)	1.20 (1.04, 1.40)	498 (130.40)	1.32 (1.17, 1.48)	292 (76.46)	1.01 (0.87, 1.18)	472 (123.59)	1.18 (1.05, 1.33)
Second trimester (N=11,158)											
No	9,506	1,043 (109.72)	Ref.	629 (66.17)	Ref.	1,008 (106.04)	Ref.	657 (69.11)	Ref.	1,003 (105.51)	Ref.
Yes	1,652	223 (134.99)	1.16 (1.00, 1.34)	158 (95.64)	1.39 (1.15, 1.67)	250 (151.33)	1.41 (1.22, 1.62)	156 (94.43)	1.30 (1.08, 1.56)	201 (121.67)	1.19 (1.03, 1.39)
Third trimester (N=12,130)											
No	10,182	1,097 (107.74)	Ref.	671 (65.90)	Ref.	1,047 (102.83)	Ref.	702 (68.95)	Ref.	1,026 (100.77)	Ref.
Yes	1,948	249 (127.82)	1.19 (1.03, 1.36)	153 (78.54)	1.22 (1.02, 1.46)	277 (142.20)	1.41 (1.23, 1.60)	171 (87.78)	1.31 (1.10, 1.56)	258 (132.44)	1.36 (1.18, 1.55)

RR, Risk Ratio; CI, Confidence Interval.

^1^Cumulative incidence was calculated as number of neurodevelopmental delay in a specific domain per 1000 children.

^2^Model adjusted for age at delivery, educational level, parity, mode of conception, pre-pregnancy body mass index, diabetic diseases, hypertensive diseases, and vaginal bleeding during early pregnancy of the mother, as well as sex and calendar year of birth of the child.

The number of mothers assessed for anxiety during the three trimesters was the same as those assessed for depression. Of these mothers, 1,742 (17.91%) mothers exhibited anxiety symptoms in the first trimester, 1,056 (9.46%) in the second trimester, and 1,314 (10.83%) in the third trimester. After multivariable adjustment, anxiety symptoms were associated with a higher risk of developmental delay in the gross motor, fine motor, problem-solving, and personal-social domains. However, we did not observe any association between prenatal anxiety and communication. Anxiety symptoms in the first, second, and third trimesters had the greatest impact on the gross motor (RR: 1.30, 95% CI: 1.09, 1.55), fine motor (RR: 1.42, 95% CI: 1.21,1.67), and personal-social development (RR: 1.38, 95% CI: 1.18, 1.61), respectively (**Table 2**). After performing multiple imputations, we found that the majority of associations remained stable. Furthermore, associations were shown between anxiety symptoms in the first trimester and delayed communication development, as well as between anxiety symptoms in the second trimester and delayed problem-solving abilities (**Supplementary Table S5**).

**Table 2:** Association between prenatal anxiety symptoms across the three trimesters of pregnancy and risk of neurodevelopmental delay among children.

Prenatal anxiety symptoms	Total (N)	Communication		Gross motor		Fine motor		Problem-solving		Personal-social	
		No. of cases (Cumulative incidence^1^)	RR (95% CI)^2^	No. of cases (Cumulative incidence^1^)	RR (95% CI)^2^	No. of cases (Cumulative incidence^1^)	RR (95% CI)^2^	No. of cases (Cumulative incidence^1^)	RR (95% CI)^2^	No. of cases (Cumulative incidence^1^)	RR (95% CI)^2^
First trimester (N=9,728)											
No	7,986	870 (108.94)	Ref.	584 (73.13)	Ref.	867 (108.56)	Ref.	567 (71.00)	Ref.	887 (111.07)	Ref.
Yes	1,742	227 (130.31)	1.15 (0.99, 1.33)	158 (90.70)	1.30 (1.09, 1.55)	234 (134.33)	1.25 (1.08, 1.44)	153 (87.83)	1.24 (1.04, 1.49)	213 (122.27)	1.05 (0.91, 1.23)
Second trimester (N=11,158)											
No	10,102	1,134 (112.25)	Ref.	695 (68.80)	Ref.	1,097 (108.59)	Ref.	720 (71.27)	Ref.	1,065 (105.42)	Ref.
Yes	1,056	132 (125.00)	1.10 (0.92, 1.32)	92 (87.12)	1.37 (1.10, 1.70)	161 (152.46)	1.42 (1.21, 1.67)	93 (88.07)	1.16 (0.93, 1.47)	139 (131.63)	1.34 (1.13, 1.59)
Third trimester (N=12,130)											
No	10,816	1,190 (110.02)	Ref.	717 (66.29)	Ref.	1,149 (106.23)	Ref.	755 (69.80)	Ref.	1,104 (102.07)	Ref.
Yes	1,314	156 (118.72)	1.08 (0.91, 1.28)	107 (81.43)	1.31 (1.06, 1.61)	175 (133.18)	1.31 (1.11, 1.53)	118 (89.80)	1.36 (1.11, 1.66)	180 (136.99)	1.38 (1.18, 1.61)

RR, Risk Ratio; CI, Confidence Interval.

^1^Cumulative incidence was calculated as number of neurodevelopmental delay in a specific domain per 1000 children.

^2^Model adjusted for age at delivery, educational level, parity, mode of conception, pre-pregnancy body mass index, diabetic diseases, hypertensive diseases, and vaginal bleeding during early pregnancy of the mother, as well as sex and calendar year of birth of the child.

**Figure 1:**
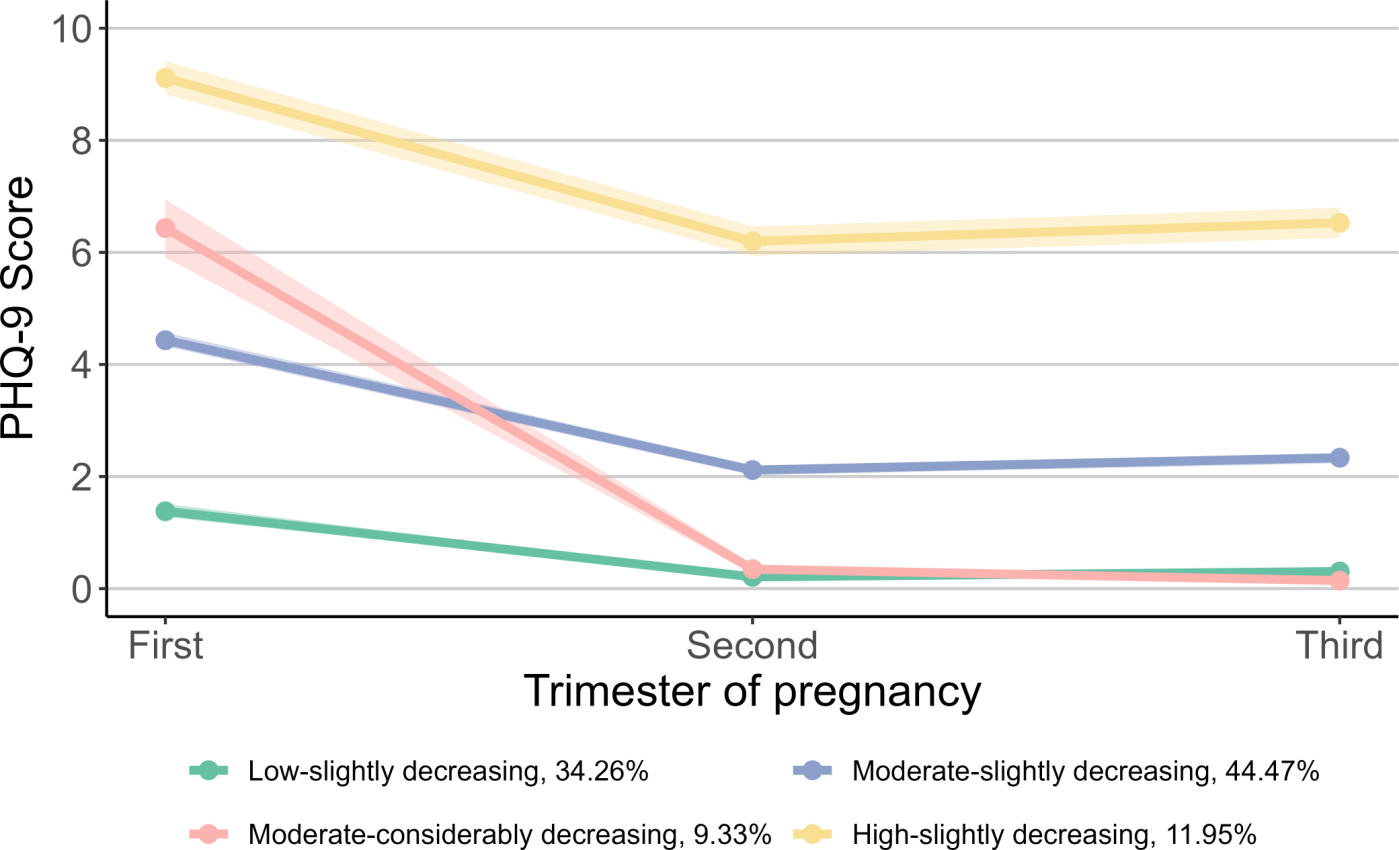
Trajectories of prenatal depressive symptoms during pregnancy. For prenatal depressive symptoms, the group proportion are as follows: 34.26% for “Low-slightly decreasing”, 44.47% for “Moderate-slightly decreasing”, 9.33% for “Moderate-considerably decreasing”, and 11.95% for “High-slightly decreasing”. PHQ-9, Patient Health Questionnaire-9.

The analysis of group-based trajectory modeling encompassed 5,893 children whose mothers completed all three assessments of prenatal depression and anxiety. **Figure 1** shows the four trajectories of prenatal depressive symptoms based on the scores of PHQ-9. The four trajectories and their proportion were as follows: 34.26% for “Low-slightly decreasing”, 44.47% for “Moderate-slightly decreasing”, 9.33% for “Moderate-considerably decreasing”, and 11.95% for “High-slightly decreasing”. The “Low-slightly decreasing” group started with a low score in the first trimester, decreased slightly in the second trimester, and remained stable afterward. Similar trends were observed in the “Moderate-slightly decreasing” and “High-slightly decreasing” groups, which started with moderate and high scores, respectively, and decreased slightly in the second and third trimesters. The “Moderate-considerably decreasing” group had a moderate score in the first trimester and decreased considerably to low scores afterward.

**Figure 2:**
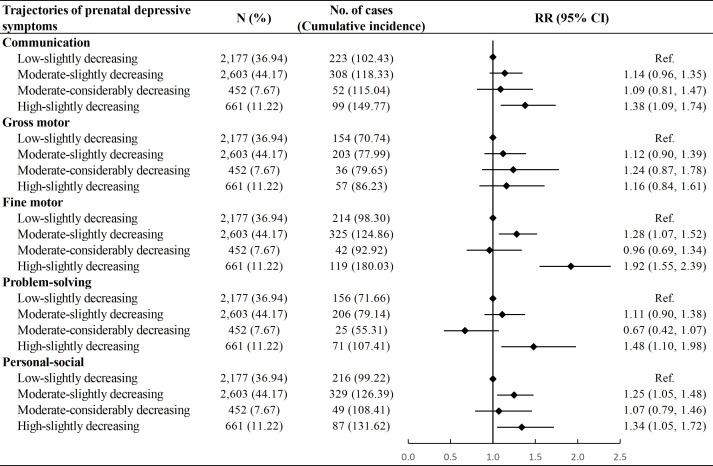
Association between trajectories of prenatal depressive symptoms and risk of neurodevelopmental delay among children. Cumulative incidence was calculated as number of neurodevelopmental delay in a specific domain per 1000 children. Model adjusted for age at delivery, educational level, parity, mode of conception, pre-pregnancy body mass index, diabetic diseases, hypertensive diseases, and vaginal bleeding during early pregnancy of the mother, as well as sex and calendar year of birth of the child. RR, Risk Ratio; CI, Confidence Interval.

Compared with children in the “Low-slightly decreasing” group, those in the “Moderate-slightly decreasing” group had a higher risk of developmental delay in the fine motor (RR: 1.28, 95% CI: 1.07, 1.52) and personal-social domains (RR: 1.25, 95% CI: 1.05, 1.48). Furthermore, children categorized within the “High-slightly decreasing” group demonstrated an even greater risk of developmental delay in communication (RR: 1.38, 95% CI: 1.09, 1.74), fine motor (RR: 1.92, 95% CI: 1.55, 2.39), problem-solving (RR: 1.48, 95% CI: 1.10, 1.98) and personal-social domains (RR: 1.34, 95% CI: 1.05, 1.72). No difference was observed between the “Low-slightly decreasing” and the “Moderate-considerably decreasing” groups (**Figure 2**). The multiple imputation analysis showed similar results (**Supplementary Table S6**).

**Figure 3:**
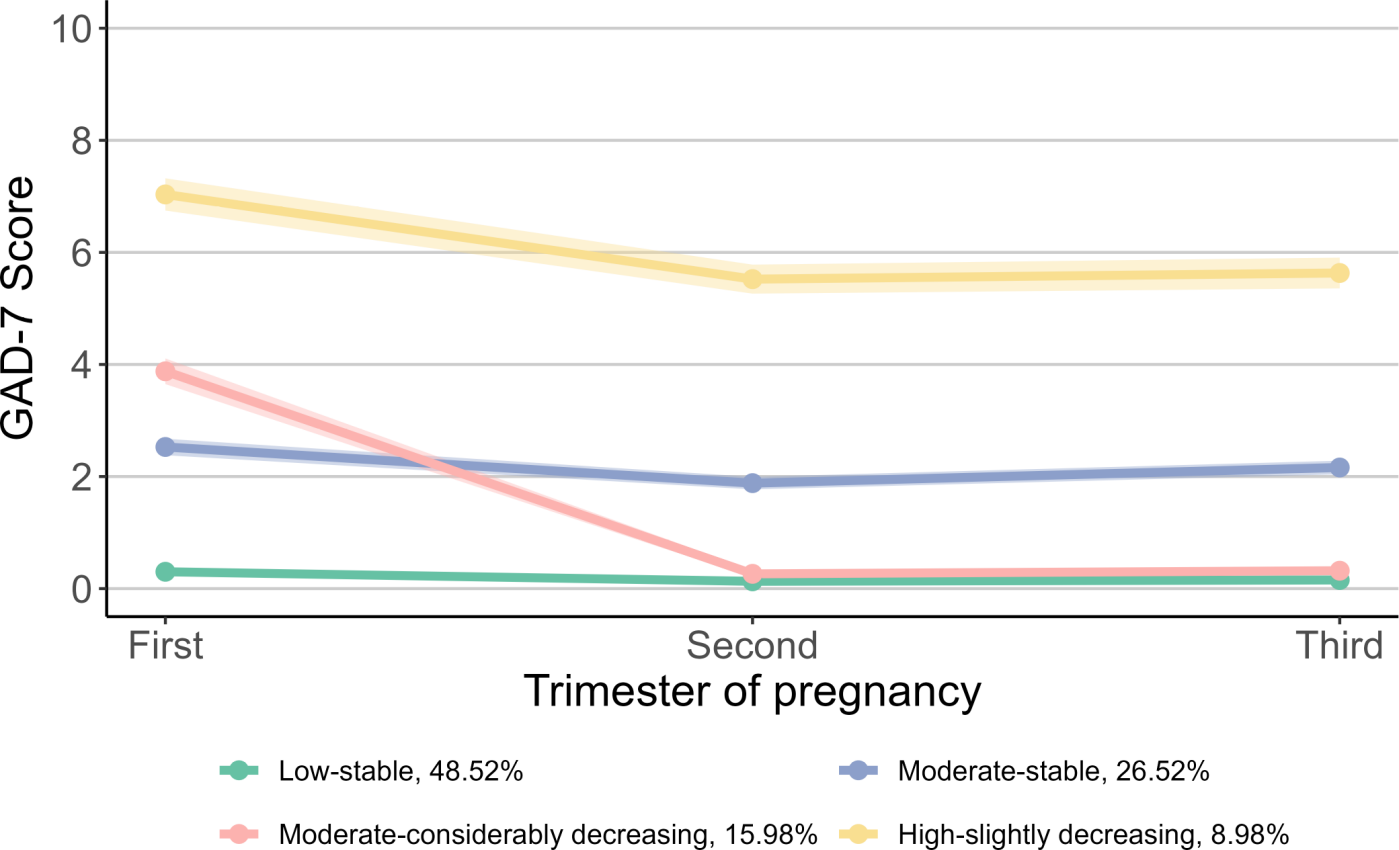
Trajectories of prenatal anxiety symptoms during pregnancy. For prenatal anxiety symptoms, the group proportion are as follows: 48.52% for “Low-stable”, 26.52% for “Moderate-stable”, 15.98% for “Moderate-considerably decreasing”, and 8.98% for “High-slightly decreasing”. GAD-7, Generalized Anxiety Disorder 7-item Scale.

Based on the scores of GAD-7, we identified four trajectory groups for prenatal anxiety symptoms: “Low-stable” group (48.52%), “Moderate-stable” group (26.52%), “Moderate-considerably decreasing” group (15.98%) and “High-slightly decreasing” group (8.98%) (**Figure 3**). The “Low-stable” and “Moderate-stable” groups consistently exhibited low and moderate scores throughout pregnancy. The “Moderate-considerably decreasing” group commenced with a moderate score, which then declined to approximately zero in the second and third trimesters. The “High-slightly decreasing” group started with a high score in the first trimester, followed by a slight decrease thereafter.

**Figure 4:**
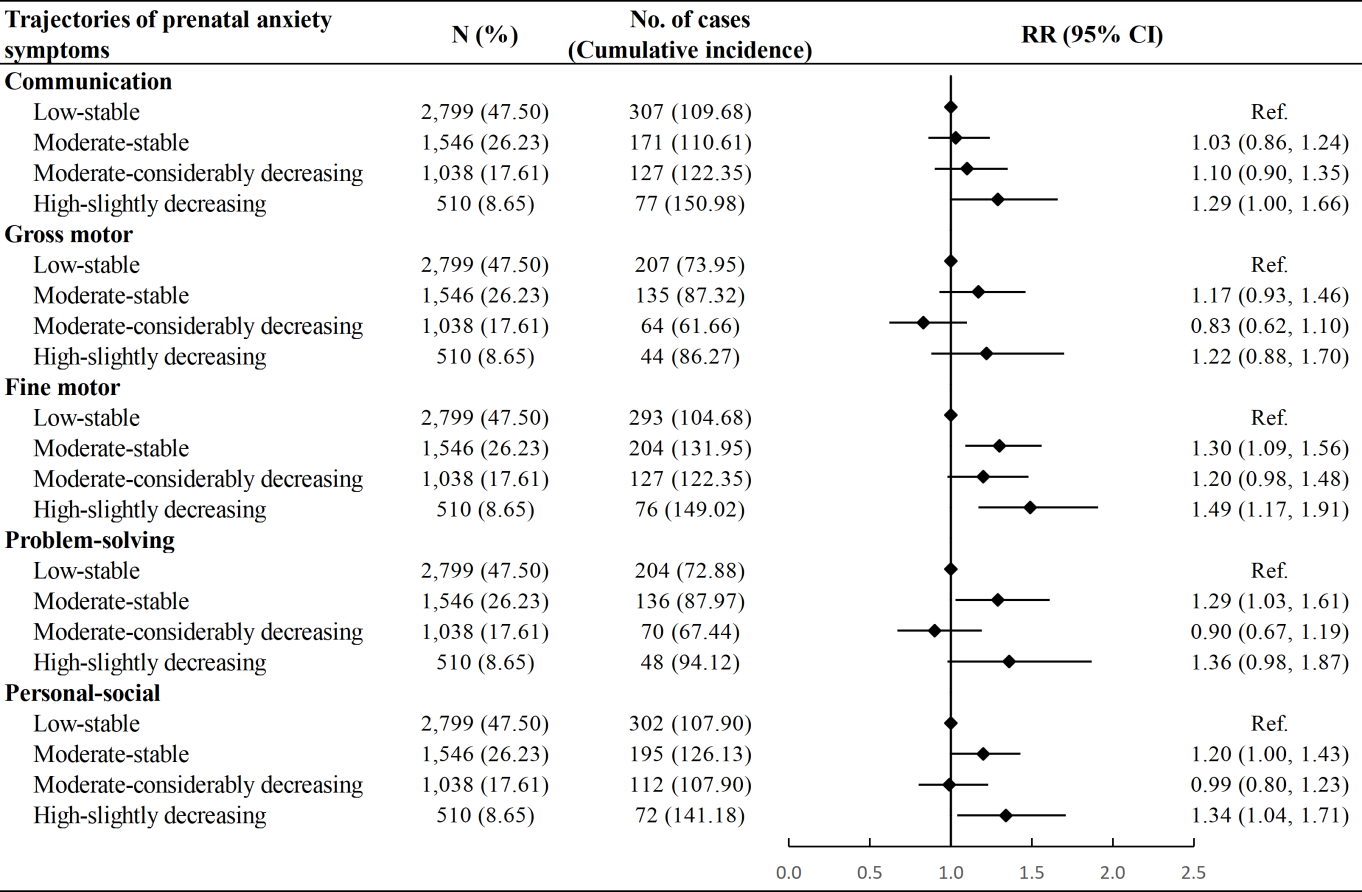
Association between trajectories of prenatal anxiety symptoms and risk of neurodevelopmental delay among children. Cumulative incidence was calculated as number of neurodevelopmental delay in a specific domain per 1000 children. Model adjusted for age at delivery, educational level, parity, mode of conception, pre-pregnancy body mass index, diabetic diseases, hypertensive diseases, and vaginal bleeding during early pregnancy of the mother, as well as sex and calendar year of birth of the child. RR, Risk Ratio; CI, Confidence Interval.

Compared with the “Low-stable” group, children in the “Moderate-stable” group showed a higher risk of delayed development in the fine motor (RR: 1.30, 95% CI: 1.09, 1.56), problem-solving (RR: 1.29, 95% CI: 1.03, 1.61) and personal-social domains (RR: 1.20, 95% CI: 1.00, 1.43), whereas children in the “High-slightly decreasing” group had a higher risk of delayed development in the communication (RR: 1.29, 95% CI: 1.00, 1.66), fine motor (RR: 1.49, 95% CI: 1.17, 1.91), and personal-socials domains (RR: 1.34, 95% CI: 1.04, 1.71). No associations were observed between the “Moderate-considerably decreasing” trajectory and the risk of neurodevelopmental delay in any domain (**Figure 4**). The associations remained stable after multiple imputations for missing values of confounders (**Supplementary Table S6**).

When stratified by children’s age at the ASQ-3 assessment, the associations of maternal psychological symptoms during each trimester and their trajectories with risk of neurodevelopmental delay were primarily observed in children aged 0-6 months. However, statistical interactions were largely insignificant (**Supplementary Tables S7–S10**). Although the associations appeared to be stronger in male children than in female children, the interaction between sex and maternal depressive or anxiety symptoms/trajectories was predominantly statistically insignificant (**Supplementary Tables S11–S14**).

## Discussion

This study found that maternal depressive and anxiety symptoms in pregnancy, especially during the second and third trimesters, were associated with a higher risk of neurodevelopmental delay in children aged 0-24 months, particularly in the domains of fine and gross motor. Four trajectories were identified respectively for prenatal depressive and anxiety symptoms. Compared with children whose mothers had low levels of psychological symptoms, those whose mothers consistently showed moderate or high levels of prenatal depressive or anxiety symptoms had an increased risk of neurodevelopmental delay, particularly in the fine motor and personal-social domains; children whose mothers experienced moderate level of depressive or anxiety symptoms only in the first trimester but decreased to low level in the second and third trimesters did not have a different risk of neurodevelopmental delay.

Our findings are in line with previous studies showing that prenatal depressive and anxiety symptoms during pregnancy were associated with a higher risk of neurodevelopmental delay. A previous prospective study in China found that maternal depressive and anxiety symptoms during mid-pregnancy were associated with delayed development of five ASQ-3 domains at age 6 and 12 months, especially impacting the fine motor domain in girls (21). Another cohort study in Australia reported that maternal anxiety symptoms during late pregnancy were associated with poorer infant social emotional and language development (31). However, a multi-center cohort study in Korea found no association between maternal depression during the first trimester of pregnancy and children’s communication, gross motor, fine motor, problem-solving, and personal-social domains at six months of age (30). Similarly, a study conducted in the US found no links between maternal depressive or anxiety symptoms at individual time points (including <20 weeks of gestation and 24-29 weeks of gestation) and cognitive outcomes in children at age 12 months (60). These differences in findings may be attributed to several reasons, including variations in the study populations, the timing and measurement of maternal psychological symptoms, and the specific neurodevelopmental outcomes assessed. By examining the impact of maternal depressive and anxiety symptoms across all three trimesters of pregnancy on the neurodevelopment in children aged 0 to 24 months, our study fills the gap in existing research and provides a more comprehensive understanding of how maternal psychological well-being throughout pregnancy influences child neurodevelopment.

The current study identified four trajectories of prenatal depressive and anxiety symptoms, which were similar to previous research (61, 62). A study from Singapore depicted four trajectories of prenatal depression, while a study from South Korea identified three trajectories of prenatal depression and four trajectories of prenatal anxiety (61, 62). The primary difference may lay in the fluctuation patterns of the trajectories, possibly due to distinct study populations with different genetic or environmental backgrounds and varying prevalence of prenatal depression and anxiety. A cohort study in China identified five trajectories for depression and anxiety from pregnancy to 42 days postpartum, with trends during the prenatal period similar to ours (23). This study corroborated our findings that children of mothers with consistently high levels of depressive or anxiety symptoms during pregnancy showed a higher risk of developmental delay in the problem-solving and personal-social domains. However, they did not find such associations for the communication and fine motor domains while we did. Additionally, they did not observe similar associations of neurodevelopmental delay with moderate levels of prenatal depressive or anxiety symptoms. These differences may stem from the different periods of child neurodevelopment assessment considered.

It is worth noting that the prevalence of depressive and anxiety symptoms was highest in early pregnancy, and depicted as an inverted J-shaped across the three trimesters of pregnancy, which was aligned with some previous studies (63, 64). This might be attributed to a combination of biological, psychological, and social factors, including hormonal fluctuations, uncertainty or concerns about the pregnancy or lifestyle changes, hyperemesis gravidarum, etc (65, 66). Nevertheless, our trajectory analysis suggested that if depressive or anxiety symptoms ameliorated during the second and third trimesters of pregnancy, the adverse impact on the child’s neurodevelopment might be mitigated. This finding underscores the importance of timely intervention and support for pregnant women encountering psychological difficulties.

When stratified by children’s age at the ASQ-3 assessment, most of the associations between maternal depressive or anxiety symptoms and children’s neurodevelopment were observed in the 0-6-month age group, consistent with previous studies (20, 21, 27). The lack of significant associations in older children may be attributed to a range of uncontrolled environmental factors that arise after birth, which could mitigate the impact of prenatal maternal factors. These factors include breastfeeding, parental care, and exposure to various environmental toxins, etc (67–70). However, these differences may also be attributed to insufficient statistical power resulting from the small sample sizes of children aged 7-12 months and 13-24 months. Therefore, larger sample sizes of studies are needed to more accurately evaluate the potential impacts of maternal mental health on the neurodevelopment in children aged 7-24 months.

The associations between maternal psychological symptoms during pregnancy and children’s neurodevelopment tended to be stronger in male children than in female children, however, most interaction effects were not statistically significant. Among the few significant interactions observed, male children demonstrated greater susceptibility to the influence of maternal prenatal depressive and anxiety symptoms. This phenomenon may be attributed to the larger biological vulnerability of male fetuses during the developmental process (71).

Prenatal depression and anxiety, considered stressors, may activate the maternal hypothalamic-pituitary-adrenal (HPA) axis, leading to elevated maternal cortisol levels, which could influence fetal brain development by altering the connectivity of brain regions such as the hippocampus and amygdala, critical for learning, memory, and emotion (72–74). These disruptions could further impair fetal neurodevelopment, aligning with our observations of domain-specific developmental delays. Besides, our findings showed that the impact of maternal depressive or anxiety symptoms during the second and third trimesters was more pronounced. Mechanistically, the placenta releases increasing amounts of corticotropin-releasing hormone from the second trimester onward, and peaking in late pregnancy, which further hyperactivates the maternal HPA axis and raises cortisol levels (75). The presence of external stressors during this period may further amplify these effects. Prolonged HPA activation and elevated cortisol levels can disrupt placental vascular formation by altering the expression of vascular endothelial growth factor and endothelial cell migration, potentially leading to placental dysplasia, compromised nutrient supply, and fetal hypoplasia, thereby exacerbating the brain development (76, 77).

The primary strengths of our study included a large sample size and a prospective study design. The collection of exposure and outcome data was conducted prospectively, effectively minimizing recall bias. Moreover, multiple assessments of prenatal depressive and anxiety symptoms allowed us to capture maternal mental health dynamics during pregnancy, to explore critical periods, and to investigate the impact of different trajectories of prenatal depressive and anxiety symptoms on children’s neurodevelopment. However, there are some limitations in our study. First, we only had data on the neurodevelopmental assessment for children up to 24 months old, limiting our ability to explore the impact of prenatal depression and anxiety on older children. Second, although we adjusted for many confounders, there were still some unadjusted factors such as maternal nutrient intake (78, 79), smoking and drinking (80), and family history of mental disorders (81–83), due to lack of data available. Third, children whose mothers underwent at least one psychological assessment during pregnancy or had ASQ-3 assessments tended to have more complete information on covariates, and their mothers were generally younger and primiparous (**Supplementary Tables S15, S16**). These factors, along with the single-hospital-based study design, may limit the generalizability of our findings to broader populations. Fourth, the proportion of mothers with depressive or anxiety symptoms among children included in the trajectory analysis was lower than that among those not included, which may have led to an underestimation of the adverse impact of maternal mental health on the children’s neurodevelopment (**Supplementary Table S17**). Additionally, the proportion of preterm birth was also lower among children included in the trajectory analysis (**Supplementary Table S18**). Given that preterm children may have a higher incidence of neurodevelopmental delays (84), this might have a modest attenuating effect on the observed associations in our study.

Our study underscores the significant influence of the dynamics of maternal psychological symptoms during pregnancy on children’s neurodevelopment, particularly highlighting the importance of maternal mental health during the mid-late stages of pregnancy. Through trajectory analysis, we demonstrated the fluctuation and plasticity of maternal depression and anxiety levels throughout pregnancy. These findings suggest that early monitoring and intervention for depression and anxiety in pregnant women could have potential benefits for both maternal well-being and child neurodevelopment. Previous randomized control trial also demonstrated that intervention for prenatal depression could be beneficial for communication and problem-solving abilities during infancy (85). Clinically, this calls for the implementation of proactive screening of psychological symptoms in early pregnancy and timely support for mothers presenting early indications of depression or anxiety.

## Conclusion

Prenatal depressive and anxiety symptoms, especially in the second and third trimesters of pregnancy, were associated with a higher risk of neurodevelopmental delay in children aged 0-24 months, especially in the fine and gross motor domains. Compared with children whose mothers had low levels of depressive or anxiety symptoms, those whose mothers experienced consistently moderate or high levels of these symptoms throughout the pregnancy had a higher risk of neurodevelopmental delay, while children whose mothers recovered to low levels of these symptoms in the second and third trimesters did not have a different risk of neurodevelopmental delay. Proactive measures are needed to enhance the mental health of pregnant women and, in turn, support optimal neurodevelopment for their offspring.

## Data Availability

The original contributions presented in the study are included in the article/**Supplementary Material**. Further inquiries can be directed to the corresponding authors.
