# Supplementary material for: Association of prenatal trajectories of depressive and anxiety symptoms with neurodevelopment of children aged 0-24 months: A prospective study
Source: Front Psychiatry. 2025 Feb 18;16:1536042. doi: 10.3389/fpsyt.2025.1536042 (PMC11876414; doi:10.3389/fpsyt.2025.1536042)
Supplement: Supplementary file 1 [file SupplementaryFile1.docx]

**Association of prenatal trajectories of depressive and anxiety symptoms with neurodevelopment of children aged 0-24 months: a prospective study**

**Supplementary materials**

**Supplementary Table S1.** Selection of the optimal number of trajectories for prenatal depressive and anxiety symptoms models

**Supplementary Table S2.** Selection of the optimal function form of trajectories for prenatal depressive and anxiety symptoms models

**Supplementary Table S3.** Characteristics of the study population

**Supplementary Table S4.** Association of prenatal depressive symptoms with risk of neurodevelopmental delay among children after multiple imputation

**Supplementary Table S5.** Association of prenatal anxiety symptoms with risk of neurodevelopmental delay among children after multiple imputation

**Supplementary Table S6.** Association between trajectories of prenatal depressive and anxiety symptoms and risk of neurodevelopmental delay among children after multiple imputation

**Supplementary Table S7.** Association between prenatal depressive symptoms across the three trimesters of pregnancy and risk of neurodevelopmental delay among children stratified by age

**Supplementary Table S8.** Association between prenatal anxiety symptoms across the three trimesters of pregnancy and risk of neurodevelopmental delay among children stratified by age

**Supplementary Table S9.** Association between trajectories of prenatal depressive symptoms and risk of neurodevelopmental delay among children stratified by age

**Supplementary Table S10.** Association between trajectories of prenatal anxiety symptoms and risk of neurodevelopmental delay among children stratified by age

**Supplementary Table S11.** Association between prenatal depressive symptoms across the three trimesters of pregnancy and risk of neurodevelopmental delay among children stratified by sex

**Supplementary Table S12.** Association between prenatal anxiety symptoms across the three trimesters of pregnancy and risk of neurodevelopmental delay among children stratified by sex

**Supplementary Table S13.** Associations between trajectories of prenatal depressive symptoms and child's neurodevelopmental delay during 0-24 months stratified by sex

**Supplementary Table S14.** Associations between trajectories of prenatal anxiety symptoms and child's neurodevelopmental delay during 0-24 months stratified by sex

**Supplementary Table S15.** Characteristics of the children with and without at least one maternal psychological assessment during pregnancy

**Supplementary Table S16.** Characteristics of the children with and without ASQ-3 assessment

**Supplementary Table S17.** Characteristics of the children with and without complete three maternal psychological assessments during pregnancy

**Supplementary Table S18.** Distribution of gestational age by completeness of maternal psychological assessment during each trimester of pregnancy

| **Supplementary Table S1. Selection of the optimal number of trajectories for prenatal depressive and anxiety symptoms models** | | | | | | | | |
| --- | --- | --- | --- | --- | --- | --- | --- | --- |
| **Number of trajectories** | **Functional form^1^** | | | | | | **BIC^2^** | **Minimum proportion of trajectory (%)** |
|  | **Trajectory 1** | **Trajectory 2** | **Trajectory 3** | **Trajectory 4** | **Trajectory 5** | **Trajectory 6** |  |  |
| **Prenatal depressive symptoms** |  |  |  |  |  |  |  |  |
| 3 | L | L | L |  |  |  | -36,044.95 | 13.75 |
| 4 | L | L | L | L |  |  | -35,418.76 | 12.43 |
| 5 | L | L | L | L | L |  | -35,024.88 | 5.05 |
| 6 | L | L | L | L | L | L | -34,754.52 | 3.98 |
| 3 | Q | Q | Q |  |  |  | -35,153.32 | 12.97 |
| 4 | Q | Q | Q | Q |  |  | -34,756.77 | 9.33 |
| 5 | Q | Q | Q | Q | Q |  | -34,378.68 | 4.37 |
| **Prenatal anxiety symptoms** |  |  |  |  |  |  |  |  |
| 3 | L | L | L |  |  |  | -27,543.47 | 10.63 |
| 4 | L | L | L | L |  |  | -26,875.06 | 9.11 |
| 5 | L | L | L | L | L |  | -26,606.56 | 5.53 |
| 6 | L | L | L | L | L | L | -26,478.92 | 2.45 |
| 3 | Q | Q | Q |  |  |  | -27,178.43 | 10.92 |
| 4 | Q | Q | Q | Q |  |  | -26,658.14 | 8.98 |
| 5 | Q | Q | Q | Q | Q |  | -26,411.25 | 4.53 |
| 6 | Q | Q | Q | Q | Q | Q | -26,302.20 | 1.78 |
| Abbreviations: BIC= Bayesian Information Criterion | | | | | | | | |
| ^1^ L and Q respectively represented a linear and quadratic functional form of trajectory. | | | | | | | | |
| ^2^ BIC was calculated by log(likelihood)-0.5*log(N)*k. N is the sample size, and k is the number of parameters in the model. | | | | | | | | |

| **Supplementary Table S2. Selection of the optimal function form of trajectories for prenatal depressive and anxiety symptoms models** | | | | | | | | | | | | | | | | | |
| --- | --- | --- | --- | --- | --- | --- | --- | --- | --- | --- | --- | --- | --- | --- | --- | --- | --- |
| **Model** | **Functional form^1^** | | | | **BIC^2^** | **Proportion of Trajectory (%)** | | | | **AvePP** | | | | **OCC^3^** | | | |
|  | **T1** | **T2** | **T3** | **T4** |  | **T1** | **T2** | **T3** | **T4** | **T1** | **T2** | **T3** | **T4** | **T1** | **T2** | **T3** | **T4** |
| **Prenatal depressive symptoms** |  |  |  |  |  |  |  |  |  |  |  |  |  |  |  |  |  |
| 4 | L | L | L | L | -35,418.76 | 23.63 | 46.06 | 17.88 | 12.43 | 0.88 | 0.91 | 0.80 | 0.91 | 26.01 | 10.89 | 17.37 | 71.16 |
| 4 | L | Q | L | Q | -34,950.93 | 23.83 | 35.03 | 29.61 | 11.54 | 0.88 | 0.80 | 0.82 | 0.89 | 23.04 | 7.41 | 10.78 | 61.44 |
| 4 | L | Q | Q | Q | -34,792.75 | 18.93 | 43.66 | 26.36 | 11.05 | 0.83 | 0.88 | 0.85 | 0.90 | 19.44 | 8.65 | 19.14 | 71.28 |
| 4 | L | L | Q | Q | -34,950.93 | 23.83 | 29.61 | 35.03 | 11.54 | 0.88 | 0.82 | 0.80 | 0.89 | 23.04 | 10.78 | 7.41 | 61.44 |
| 4 | Q | Q | Q | Q | -34,756.77 | 34.26 | 44.47 | 9.33 | 11.95 | 0.87 | 0.90 | 0.78 | 0.91 | 11.17 | 11.51 | 41.58 | 81.80 |
| **Prenatal anxiety symptoms** |  |  |  |  |  |  |  |  |  |  |  |  |  |  |  |  |  |
| 4 | L | L | L | L | -26,875.06 | 47.38 | 15.39 | 28.12 | 9.11 | 0.94 | 0.81 | 0.87 | 0.92 | 17.83 | 24.18 | 16.00 | 125.93 |
| 4 | L | Q | L | Q | -26,703.51 | 47.19 | 19.87 | 23.72 | 9.22 | 0.94 | 0.82 | 0.83 | 0.92 | 16.98 | 18.76 | 15.13 | 114.20 |
| 4 | L | Q | Q | Q | -26,676.81 | 47.24 | 26.51 | 17.35 | 8.90 | 0.94 | 0.86 | 0.79 | 0.91 | 17.25 | 17.84 | 18.30 | 109.08 |
| 4 | L | L | Q | Q | -26,703.51 | 47.19 | 23.72 | 19.87 | 9.22 | 0.94 | 0.83 | 0.82 | 0.92 | 16.98 | 15.13 | 18.76 | 114.20 |
| 4 | Q | Q | Q | Q | -26,658.14 | 48.52 | 26.52 | 15.98 | 8.98 | 0.95 | 0.87 | 0.75 | 0.92 | 21.25 | 18.39 | 13.68 | 117.56 |
| Abbreviations: T=Trajectory, BIC= Bayesian Information Criterion, AvePP=Average posterior probabilities, OCC=Odds of correct classification | | | | | | | | | | | | | | | | | |
| ^1^ L and Q respectively represented a linear and quadratic functional form of trajectory. | | | | | | | | |  |  |  |  |  |  |  |  |  |
| ^2^ BIC was calculated by log(likelihood)-0.5*log(N)*k. N is the sample size, and k is the number of parameters in the model. | | | | | | | | | | | | | | | | | |
| ^3^ OCC was calculated by [AvePP/(1-AvePP)]/[π /(1-π)], π is the estimated posterior probability. | | | | | | | | | | | | | | | | | |

| **Supplementary Table S3. Characteristics of the study population** | | | | | | | | | | | | | | | |
| --- | --- | --- | --- | --- | --- | --- | --- | --- | --- | --- | --- | --- | --- | --- | --- |
| **Characteristics** | | **Overall (N=16,229) (N [%])** | | **Neurodevelopmental delay (No. [cumulative incidence^1^])** | | | | | | | | | | |  |
|  |  |  |  | **Communication  (N=1,791)** | | **Gross motor (N=1,127)** | | **Fine motor (N=1,750)** | | **Problem-solving (N=1,137)** | | **Personal-social (N=1,724)** | | |  |
| **Mothers** | |  | |  | |  | |  | |  | |  | |  |  |
| **Age at delivery (years)** | |  | |  | |  | |  | |  | |  | |  |  |
| As continuous variable  (mean [SD]) | | 30.35 (3.76) | | 30.45 (3.84) | | 30.48 (3.64) | | 30.53 (3.83) | | 30.57 (3.87) | | 30.43 (3.76) | | |  |
| <25 | | 1,297 (7.99) | | 150 (115.65) | | 78 (60.14) | | 133 (102.54) | | 88 (67.85) | | 130 (100.23) | | |  |
| 25-29 | | 5,792 (35.69) | | 610 (105.32) | | 383 (66.13) | | 574 (99.10) | | 371 (64.05) | | 586 (101.17) | | |  |
| 30-34 | | 6,958 (42.87) | | 787 (113.11) | | 525 (75.45) | | 800 (114.98) | | 511 (73.44) | | 784 (112.68) | | |  |
| ≥35 | | 2,182 (13.45) | | 244 (111.82) | | 141 (64.62) | | 243 (111.37) | | 167 (76.54) | | 224 (102.66) | | |  |
| **Educational level** | |  | |  | |  | |  | |  | |  | |  |  |
| Senior high school or below | | 2,464 (15.18) | | 309 (125.41) | | 155 (62.91) | | 285 (115.67) | | 178 (72.24) | | 237 (96.19) | | |  |
| Undergraduate | | 12,267 (75.59) | | 1,306 (106.46) | | 869 (70.84) | | 1,298 (105.81) | | 822 (67.01) | | 1,298 (105.81) | | |  |
| Graduate | | 1,262 (7.78) | | 151 (119.65) | | 79 (62.60) | | 140 (110.94) | | 112 (88.75) | | 160 (126.78) | | |  |
| Missing | | 236 (1.45) | | 25 (105.93) | | 24 (101.69) | | 27 (114.41) | | 25 (105.93) | | 29 (122.88) | | |  |
| **Parity** | |  | |  | |  | |  | |  | |  | |  |  |
| 1 | | 11,302 (69.64) | | 1,326 (117.32) | | 778 (68.84) | | 1247 (110.33) | | 802 (70.96) | | 1,253 (110.87) | | |  |
| 2 | | 4,347 (26.79) | | 393 (90.41) | | 303 (69.70) | | 438 (100.76) | | 289 (66.48) | | 415 (95.47) | | |  |
| ≥3 | | 580 (3.57) | | 72 (124.14) | | 46 (79.31) | | 65 (112.07) | | 46 (79.31) | | 56 (96.55) | | |  |
| **Mode of conception** | |  | |  | |  | |  | |  | |  | |  |  |
| Naturally conceived | | 15,189 (93.59) | | 1,677 (110.41) | | 1,048 (69.00) | | 1,625 (106.99) | | 1,048 (69.00) | | 1,601 (105.41) | | |  |
| ART | | 810 (4.99) | | 90 (111.11) | | 56 (69.14) | | 98 (120.99) | | 64 (79.01) | | 95 (117.28) | | |  |
| Missing | | 230 (1.42) | | 24 (104.35) | | 23 (100.00) | | 27 (117.39) | | 25 (108.70) | | 28 (121.74) | | |  |
| **Pre-pregnancy BMI** | |  | |  | |  | |  | |  | |  | |  |  |
| As continuous variable  (mean [SD]) | | 20.93 (2.78) | | 20.98 (2.78) | | 20.94 (2.83) | | 20.94 (2.82) | | 21.01 (2.74) | | 20.97 (2.76) | | |  |
| <18.5 | | 2,813 (17.33) | | 298 (105.94) | | 193 (68.61) | | 310 (110.20) | | 198 (70.39) | | 290 (103.09) | | |  |
| 18.5-23.9 | | 11,131 (68.59) | | 1,242 (111.58) | | 759 (68.19) | | 1,200 (107.81) | | 774 (69.54) | | 1,193 (107.18) | | |  |
| 24.0-27.9 | | 1,734 (10.68) | | 189 (109.00) | | 127 (73.24) | | 183 (105.54) | | 119 (68.63) | | 178 (102.65) | | |  |
| ≥28.0 | | 312 (1.92) | | 36 (115.38) | | 24 (76.92) | | 29 (92.95) | | 20 (64.10) | | 34 (108.97) | | |  |
| Missing | | 239 (1.47) | | 26 (108.79) | | 24 (100.42) | | 28 (117.15) | | 26 (108.79) | | 29 (121.34) | | |  |
| **Diabetic diseases** | |  | |  | |  | |  | |  | |  | |  |  |
| No | | 12,685 (78.16) | | 1,397 (110.13) | | 878 (69.22) | | 1,344 (105.95) | | 875 (68.98) | | 1,337 (105.40) | | |  |
| Yes | | 3,544 (21.84) | | 394 (111.17) | | 249 (70.26) | | 406 (114.56) | | 262 (73.93) | | 387 (109.20) | | |  |
| **Hypertensive diseases** | |  | |  | |  | |  | |  | |  | |  |  |
| No | | 14,900 (91.81) | | 1,637 (109.87) | | 1,030 (69.13) | | 1,578 (105.91) | | 1047 (70.27) | | 1,543 (103.56) | | |  |
| Yes | | 1,329 (8.19) | | 154 (115.88) | | 97 (72.99) | | 172 (129.42) | | 90 (67.72) | | 181 (136.19) | | |  |
| **Vaginal bleeding during early pregnancy** | |  | |  | |  | |  | |  | |  | |  |  |
| No | | 11,731 (72.28) | | 1,282 (109.28) | | 816 (69.56) | | 1,225 (104.42) | | 807 (68.79) | | 1,226 (104.51) | | |  |
| Yes | | 2,641 (16.27) | | 308 (116.62) | | 170 (64.37) | | 334 (126.47) | | 193 (73.08) | | 311 (117.76) | | |  |
| Missing | | 1,857 (11.44) | | 201 (108.24) | | 141 (75.93) | | 191 (102.85) | | 137 (73.77) | | 187 (100.70) | | |  |
| **Children** | |  | |  | |  | |  | |  | |  | |  |  |
| **Sex** | |  | |  | |  | |  | |  | |  | |  |  |
| Male | | 8,731 (53.80) | | 1,007 (115.34) | | 608 (69.64) | | 954 (109.27) | | 624 (71.47) | | 983 (112.59) | | |  |
| Female | | 7,498 (46.20) | | 784 (104.56) | | 519 (69.22) | | 796 (106.16) | | 513 (68.42) | | 741 (98.83) | | |  |
| **Gestational age at birth (weeks)** | |  | |  | |  | |  | |  | |  | |  |  |
| As continuous variable  (mean [SD]) | | 38.85 (1.33) | | 38.59 (1.55) | | 38.67 (1.43) | | 38.58 (1.38) | | 38.56 (1.51) | | 38.52 (1.55) | | |  |
| ≤33 | | 89 (0.55) | | 26 (292.13) | | 12 (134.83) | | 14 (157.30) | | 14 (157.30) | | 28 (314.61) | | |  |
| 34-36 | | 615 (3.79) | | 98 (159.35) | | 38 (61.79) | | 81 (131.71) | | 44 (71.54) | | 79 (128.46) | | |  |
| 37-38 | | 4,660 (28.71) | | 575 (123.39) | | 392 (84.12) | | 641 (137.55) | | 422 (90.56) | | 621 (133.26) | | |  |
| 39-40 | | 9,819 (60.50) | | 1,008 (102.66) | | 632 (64.37) | | 938 (95.53) | | 616 (62.74) | | 916 (93.29) | | |  |
| 41 | | 1,046 (6.45) | | 84 (80.31) | | 53 (50.67) | | 76 (72.66) | | 41 (39.20) | | 80 (76.48) | | |  |
| **Birth weight (grams)** | |  | |  | |  | |  | |  | |  | | |  |
| As continuous variable  (mean [SD]) | | 3,217.76 (411.54) | | 3,150.77 (451.27) | | 3,169.68 (426.80) | | 3,158.20 (431.68) | | 3,156.52  (439.42) | | 3,147.61  (456.73) | | |  |
| <2,500 | | 577 (3.56) | | 121 (209.71) | | 52 (90.12) | | 100 (173.31) | | 65 (112.65) | | 112 (194.11) | | |  |
| 2,500-2,999 | | 3,684 (22.70) | | 437 (118.62) | | 291 (78.99) | | 464 (125.95) | | 286 (77.63) | | 436 (118.35) | | |  |
| 3,000-3,499 | | 7,907 (48.72) | | 857 (108.38) | | 544 (68.80) | | 816 (103.20) | | 541 (68.42) | | 816 (103.20) | | |  |
| 3,500-3,999 | | 3,535 (21.78) | | 331 (93.64) | | 205 (57.99) | | 316 (89.39) | | 218 (61.67) | | 311 (87.98) | | |  |
| ≥4,000 | | 526 (3.24) | | 45 (85.55) | | 35 (66.54) | | 54 (102.66) | | 27 (51.33) | | 49 (93.16) | | |  |
| **Calendar year of birth** | |  | |  | |  | |  | |  | |  | | |  |
| 2020 | | 1,631 (10.05) | | 160 (98.10) | | 86 (52.73) | | 171 (104.84) | | 105 (64.38) | | 160 (98.10) | | |  |
| 2021 | | 4,990 (30.75) | | 494 (99.00) | | 341 (68.34) | | 444 (88.98) | | 288 (57.72) | | 472 (94.59) | | |  |
| 2022 | | 5,487 (33.81) | | 626 (114.09) | | 392 (71.44) | | 630 (114.82) | | 401 (73.08) | | 596 (108.62) | | |  |
| 2023 | | 4,121 (25.39) | | 511 (124.00) | | 308 (74.74) | | 505 (122.54) | | 343 (83.23) | | 496 (120.36) | | |  |
| Abbreviations: SD=Standard Deviation; ART=Assisted Reproductive Technology; BMI=Body Mass Index. | | | | | | | | | | | | | | |  |
| ^1^ Cumulative incidence was calculated as number of neurodevelopmental delays in a specific domain per 1000 children. | | | | | | | | | | | | | | |  |

| **Supplementary Table S4. Association of prenatal depressive symptoms with risk of neurodevelopmental delay among children after multiple imputation** | | | | | |
| --- | --- | --- | --- | --- | --- |
| **Prenatal depressive symptoms** | **RR (95% CI)^1^** | | | | |
|  | **Communication** | **Gross motor** | **Fine motor** | **Problem-solving** | **Personal-social** |
| **First trimester (N=9,728)** |  |  |  |  |  |
| No | Ref. | Ref. | Ref. | Ref. | Ref. |
| Yes | 1.20 (1.07, 1.34) | 1.18 (1.03, 1.36) | 1.30 (1.16, 1.45) | 1.08 (0.93, 1.25) | 1.18 (1.06, 1.33) |
| **Second trimester (N=11,158)** |  |  |  |  |  |
| No | Ref. | Ref. | Ref. | Ref. | Ref. |
| Yes | 1.24 (1.08, 1.42) | 1.49 (1.26, 1.76) | 1.46 (1.28, 1.66) | 1.42 (1.20, 1.68) | 1.19 (1.03, 1.37) |
| **Third trimester (N=12,130)** |  |  |  |  |  |
| No | Ref. | Ref. | Ref. | Ref. | Ref. |
| Yes | 1.21 (1.06, 1.38) | 1.24 (1.04, 1.46) | 1.42 (1.26, 1.61) | 1.33 (1.13, 1.56) | 1.36 (1.20, 1.55) |
| Abbreviations: RR=Risk Ratio; CI= Confidence Interval. | | | | | |
| \| ^1^ Model adjusted for maternal age at delivery, maternal educational level, parity, mode of conception, pre-pregnancy body mass index, diabetic diseases, hypertensive diseases, vaginal bleeding during early pregnancy, as well as sex and calendar year of birth of the child. \| \| --- \| | | | | | |

| **Supplementary Table S5. Association of prenatal anxiety symptoms with risk of neurodevelopmental delay among children after multiple imputation** | | | | | |
| --- | --- | --- | --- | --- | --- |
| **Prenatal anxiety symptoms** | **RR (95% CI)^1^** | | | | |
|  | **Communication** | **Gross motor** | **Fine motor** | **Problem-solving** | **Personal-social** |
| **First trimester (N=9,728)** |  |  |  |  |  |
| No | Ref. | Ref. | Ref. | Ref. | Ref. |
| Yes | 1.20 (1.04, 1.37) | 1.24 (1.05, 1.47) | 1.24 (1.08, 1.42) | 1.25 (1.06, 1.49) | 1.10 (0.96, 1.27) |
| **Second trimester (N=11,158)** |  |  |  |  |  |
| No | Ref. | Ref. | Ref. | Ref. | Ref. |
| Yes | 1.12 (0.94, 1.32) | 1.30 (1.06, 1.60) | 1.43 (1.23, 1.67) | 1.26 (1.02, 1.56) | 1.27 (1.08, 1.50) |
| **Third trimester (N=12,130)** |  |  |  |  |  |
| No | Ref. | Ref. | Ref. | Ref. | Ref. |
| Yes | 1.11 (0.95, 1.30) | 1.28 (1.05, 1.56) | 1.28 (1.10, 1.49) | 1.35 (1.12, 1.63) | 1.38 (1.19, 1.59) |
| Abbreviations: RR=Risk Ratio; CI= Confidence Interval. | | | | | |
| ^1^ Model adjusted for maternal age at delivery, maternal educational level, parity, mode of conception, pre-pregnancy body mass index, diabetic diseases, hypertensive diseases, vaginal bleeding during early pregnancy, as well as sex and calendar year of birth of the child. | | | | | |

| **Supplementary Table S6. Association between trajectories of prenatal depressive and anxiety symptoms and risk of neurodevelopmental delay among children after multiple imputation** | | | | | |
| --- | --- | --- | --- | --- | --- |
| **Trajectories** | **RR (95% CI)^1^** | | | | |
|  | **Communication** | **Gross motor** | **Fine motor** | **Problem-solving** | **Personal-social** |
| **Prenatal depressive symptoms** |  |  |  |  |  |
| Low-slightly decreasing | Ref. | Ref. | Ref. | Ref. | Ref. |
| Moderate-slightly decreasing | 1.15 (0.98, 1.35) | 1.11 (0.91, 1.36) | 1.27 (1.08, 1.49) | 1.11 (0.91, 1.36) | 1.27 (1.08, 1.49) |
| Moderate-considerably decreasing | 1.10 (0.83, 1.46) | 1.14 (0.81, 1.62) | 0.94 (0.68, 1.29) | 0.78 (0.52, 1.17) | 1.09 (0.81, 1.46) |
| High-slightly decreasing | 1.45 (1.16, 1.80) | 1.24 (0.92, 1.67) | 1.86 (1.51, 2.29) | 1.56 (1.19, 2.04) | 1.35 (1.07, 1.71) |
| **Prenatal anxiety symptoms** |  |  |  |  |  |
| Low-stable | Ref. | Ref. | Ref. | Ref. | Ref. |
| Moderate-stable | 1.00 (0.84, 1.20) | 1.21 (0.98, 1.49) | 1.27 (1.07, 1.50) | 1.25 (1.01, 1.54) | 1.16 (0.98, 1.38) |
| Moderate-considerably decreasing | 1.09 (0.90, 1.33) | 0.84 (0.64, 1.10) | 1.15 (0.95, 1.40) | 0.93 (0.71, 1.21) | 0.99 (0.81, 1.22) |
| High-slightly decreasing | 1.35 (1.07, 1.71) | 1.19 (0.87, 1.63) | 1.43 (1.13, 1.81) | 1.35 (1.00, 1.82) | 1.31 (1.03, 1.66) |
| Abbreviations: RR=Risk Ratio; CI= Confidence Interval. | | | | | |
| ^1^ Model adjusted for age at delivery, educational level, parity, mode of conception, pre-pregnancy body mass index, diabetic diseases, hypertensive diseases, vaginal bleeding during early pregnancy, as well as sex and calendar year of birth of the child. | | | | | |

| **Supplementary Table S7. Association between prenatal depressive symptoms across the three trimesters of pregnancy and risk of neurodevelopmental delay among children stratified by age** | | | | | | |
| --- | --- | --- | --- | --- | --- | --- |
| **Prenatal depressive symptoms** | **Total (N)** | **RR (95% CI)^1^** | | | | |
|  |  | **Communication** | **Gross motor** | **Fine motor** | **Problem-solving** | **Personal-social** |
| **First trimester** |  |  |  |  |  |  |
| 0-6 months | 7,335 | 1.16 (1.01, 1.34) | 1.25 (1.04, 1.49) | 1.33 (1.17, 1.52) | 1.05 (0.88, 1.24) | 1.19 (1.04, 1.37) |
| 7-12 months | 1,673 | 1.30 (0.92, 1.82) | 1.09 (0.79, 1.51) | 1.79 (1.08, 3.00) | 1.14 (0.63, 2.03) | 1.12 (0.75, 1.67) |
| 13-24 months | 720 | 1.44 (0.89, 2.34) | 1.08 (0.56, 2.06) | 0.93 (0.50, 1.70) | 0.74 (0.41, 1.29) | 1.26 (0.74, 2.16) |
| *P* for interaction |  | 0.594 | 0.722 | 0.248 | 0.454 | 0.933 |
| **Second trimester** |  |  |  |  |  |  |
| 0-6 months | 8,539 | 1.15 (0.96, 1.37) | 1.58 (1.26, 1.96) | 1.43 (1.21, 1.67) | 1.45 (1.18, 1.77) | 1.25 (1.05, 1.49) |
| 7-12 months | 1,837 | 1.32 (0.87, 1.93) | 0.90 (0.55, 1.40) | 1.84 (1.05, 3.08) | 0.90 (0.39, 1.79) | 1.03 (0.59, 1.68) |
| 13-24 months | 782 | 0.93 (0.47, 1.68) | 1.27 (0.57, 2.56) | 0.73 (0.32, 1.45) | 0.38 (0.11, 0.93) | 0.76 (0.35, 1.47) |
| *P* for interaction |  | 0.611 | 0.075 | 0.117 | 0.019 | 0.307 |
| **Third trimester** |  |  |  |  |  |  |
| 0-6 months | 8,943 | 1.20 (1.01, 1.42) | 1.29 (1.02, 1.61) | 1.41 (1.20, 1.64) | 1.35 (1.10, 1.65) | 1.36 (1.15, 1.59) |
| 7-12 months | 2,138 | 1.21 (0.82, 1.74) | 1.03 (0.67, 1.54) | 1.27 (0.70, 2.18) | 1.38 (0.74, 2.42) | 1.33 (0.83, 2.05) |
| 13-24 months | 1,049 | 1.01 (0.58, 1.67) | 1.17 (0.55, 2.27) | 1.54 (0.89, 2.56) | 0.94 (0.48, 1.70) | 1.39 (0.81, 2.29) |
| *P* for interaction |  | 0.808 | 0.621 | 0.877 | 0.524 | 0.990 |
| Abbreviations: RR=Risk Ratio; CI= Confidence Interval. | | | | | | |
| ^1^ Model adjusted for age at delivery, educational level, parity, mode of conception, pre-pregnancy body mass index, diabetic diseases, hypertensive diseases, and vaginal bleeding during early pregnancy of the mother, as well as sex and calendar year of birth of the child. | | | | | | |

| **Supplementary Table S8. Association between prenatal anxiety symptoms across the three trimesters of pregnancy and risk of neurodevelopmental delay among children stratified by age** | | | | | | |
| --- | --- | --- | --- | --- | --- | --- |
| **Prenatal anxiety symptoms** | **Total (N)** | **RR (95% CI)^1^** | | | | |
|  |  | **Communication** | **Gross motor** | **Fine motor** | **Problem-solving** | **Personal-social** |
| **First trimester** |  |  |  |  |  |  |
| 0-6 months | 7,335 | 1.16 (0.97, 1.38) | 1.50 (1.21, 1.85) | 1.22 (1.03, 1.43) | 1.28 (1.04, 1.57) | 1.10 (0.92, 1.30) |
| 7-12 months | 1,673 | 1.10 (0.71, 1.65) | 0.87 (0.55, 1.31) | 1.17 (0.61, 2.10) | 0.90 (0.39, 1.84) | 0.59 (0.31, 1.03) |
| 13-24 months | 720 | 1.07 (0.58, 1.85) | 0.93 (0.38, 2.01) | 2.13 (1.10, 3.94) | 1.18 (0.59, 2.18) | 1.46 (0.78, 2.60) |
| *P* for interaction |  | 0.935 | 0.044 | 0.211 | 0.661 | 0.074 |
| **Second trimester** |  |  |  |  |  |  |
| 0-6 months | 8,539 | 1.09 (0.87, 1.35) | 1.33 (1.00, 1.73) | 1.38 (1.13, 1.66) | 1.27 (0.98, 1.63) | 1.40 (1.14, 1.70) |
| 7-12 months | 1,837 | 1.45 (0.91, 2.21) | 1.37 (0.84, 2.13) | 1.80 (0.95, 3.18) | 0.77 (0.27, 1.75) | 1.12 (0.60, 1.93) |
| 13-24 months | 782 | 0.51 (0.15, 1.23) | 1.76 (0.71, 3.76) | 1.79 (0.85, 3.41) | 0.69 (0.21, 1.70) | 1.07 (0.44, 2.21) |
| *P* for interaction |  | 0.129 | 0.794 | 0.532 | 0.313 | 0.637 |
| **Third trimester** |  |  |  |  |  |  |
| 0-6 months | 8,943 | 1.17 (0.95, 1.42) | 1.36 (1.04, 1.74) | 1.31 (1.08, 1.57) | 1.36 (1.07, 1.71) | 1.40 (1.16, 1.69) |
| 7-12 months | 2,138 | 1.14 (0.72, 1.73) | 1.25 (0.77, 1.92) | 1.86 (1.02, 3.19) | 1.54 (0.76, 2.83) | 1.28 (0.74, 2.08) |
| 13-24 months | 1,049 | 0.42 (0.16, 0.89) | 1.13 (0.49, 2.28) | 1.00 (0.50, 1.83) | 1.21 (0.62, 2.19) | 1.34 (0.73, 2.29) |
| *P* for interaction |  | 0.058 | 0.867 | 0.320 | 0.866 | 0.933 |
| Abbreviations: RR=Risk Ratio; CI= Confidence Interval. | | | | | | |
| ^1^ Model adjusted for age at delivery, educational level, parity, mode of conception, pre-pregnancy body mass index, diabetic diseases, hypertensive diseases, and vaginal bleeding during early pregnancy of the mother, as well as sex and calendar year of birth of the child. | | | | | | |

| **Supplementary Table S9. Association between trajectories of prenatal depressive symptoms and risk of neurodevelopmental delay among children stratified by age** | | | | | | | | |
| --- | --- | --- | --- | --- | --- | --- | --- | --- |
| **Trajectories of prenatal depressive symptoms** | **Total (N)** | **0-6 months** | | **7-12 months** | | **13-24 months** | | ***P* for interaction** |
|  |  | **N (%)** | **RR (95% CI)^1^** | **N (%)** | **RR (95% CI)^1^** | **N (%)** | **RR (95% CI)^1^** |  |
| **Communication** |  |  |  |  |  |  |  |  |
| Low-slightly decreasing | 2,177 | 1,847 (37.56) | Ref. | 268 (34.27) | Ref. | 62 (32.12) | Ref. |  |
| Moderate-slightly decreasing | 2,603 | 2,146 (43.64) | 1.10 (0.92, 1.32) | 359 (45.91) | 1.56 (0.88, 2.76) | 98 (50.78) | 1.48 (0.40, 5.49) | 0.492 |
| Moderate-considerably decreasing | 452 | 385 (7.83) | 1.10 (0.81, 1.50) | 62 (7.93) | 0.79 (0.24, 2.57) | 5 (2.59) | 3.55 (0.43, 29.25) | 0.475 |
| High-slightly decreasing | 661 | 540 (10.98) | 1.34 (1.05, 1.72) | 93 (11.89) | 1.89 (0.90, 3.98) | 28 (14.51) | 1.41 (0.26, 7.64) | 0.694 |
| **Gross motor** |  |  |  |  |  |  |  |  |
| Low-slightly decreasing | 2,177 | 1,847 (37.56) | Ref. | 268 (34.27) | Ref. | 62 (32.12) | Ref. |  |
| Moderate-slightly decreasing | 2,603 | 2,146 (43.64) | 1.06 (0.84, 1.35) | 359 (45.91) | 1.21 (0.71, 2.04) | 98 (50.78) | 2.89 (0.65, 12.76) | 0.403 |
| Moderate-considerably decreasing | 452 | 385 (7.83) | 0.95 (0.61, 1.48) | 62 (7.93) | 2.86 (1.49, 5.48) | 5 (2.59) | NA | 0.006 |
| High-slightly decreasing | 661 | 540 (10.98) | 1.16 (0.81, 1.67) | 93 (11.89) | 1.17 (0.54, 2.57) | 28 (14.51) | 1.23 (0.12, 12.91) | 0.999 |
| **Fine motor** |  |  |  |  |  |  |  |  |
| Low-slightly decreasing | 2,177 | 1,847 (37.56) | Ref. | 268 (34.27) | Ref. | 62 (32.12) | Ref. |  |
| Moderate-slightly decreasing | 2,603 | 2,146 (43.64) | 1.27 (1.06, 1.51) | 359 (45.91) | 2.01 (0.80, 5.05) | 98 (50.78) | 1.25 (0.40, 3.91) | 0.625 |
| Moderate-considerably decreasing | 452 | 385 (7.83) | 0.94 (0.67, 1.32) | 62 (7.93) | 1.41 (0.29, 6.76) | 5 (2.59) | 2.92 (0.37, 22.75) | 0.508 |
| High-slightly decreasing | 661 | 540 (10.98) | 1.79 (1.43, 2.26) | 93 (11.89) | 4.70 (1.74, 12.73) | 28 (14.51) | 3.20 (0.99, 10.38) | 0.125 |
| **Problem solving** |  |  |  |  |  |  |  |  |
| Low-slightly decreasing | 2,177 | 1,847 (37.56) | Ref. | 268 (34.27) | Ref. | 62 (32.12) | Ref. |  |
| Moderate-slightly decreasing | 2,603 | 2,146 (43.64) | 1.09 (0.88, 1.37) | 359 (45.91) | 1.98 (0.79, 4.99) | 98 (50.78) | 0.74 (0.24, 2.31) | 0.360 |
| Moderate-considerably decreasing | 452 | 385 (7.83) | 0.65 (0.40, 1.06) | 62 (7.93) | 0.77 (0.09, 6.29) | 5 (2.59) | 2.46 (0.34, 18.04) | 0.445 |
| High-slightly decreasing | 661 | 540 (10.98) | 1.54 (1.14, 2.07) | 93 (11.89) | 1.04 (0.21, 5.09) | 28 (14.51) | 0.89 (0.18, 4.51) | 0.734 |
| **Personal-social** |  |  |  |  |  |  |  |  |
| Low-slightly decreasing | 2,177 | 1,847 (37.56) | Ref. | 268 (34.27) | Ref. | 62 (32.12) | Ref. |  |
| Moderate-slightly decreasing | 2,603 | 2,146 (43.64) | 1.28 (1.07, 1.53) | 359 (45.91) | 1.14 (0.62, 2.08) | 98 (50.78) | 1.01 (0.35, 2.89) | 0.859 |
| Moderate-considerably decreasing | 452 | 385 (7.83) | 1.14 (0.83, 1.57) | 62 (7.93) | 0.53 (0.12, 2.27) | 5 (2.59) | NA | 0.312 |
| High-slightly decreasing | 661 | 540 (10.98) | 1.34 (1.03, 1.74) | 93 (11.89) | 0.97 (0.37, 2.56) | 28 (14.51) | 2.37 (0.74, 7.55) | 0.510 |
| Abbreviations: RR=Risk Ratio; CI= Confidence Interval; NA=Not available. | | | | | | | | |
| ^1^ Model adjusted for age at delivery, educational level, parity, mode of conception, pre-pregnancy body mass index, diabetic diseases, hypertensive diseases, and vaginal bleeding during early pregnancy of the mother, as well as sex and calendar year of birth of the child. | | | | | | | | |

| **Supplementary Table S10. Association between trajectories of prenatal anxiety symptoms and risk of neurodevelopmental delay among children stratified by age** | | | | | | | | |
| --- | --- | --- | --- | --- | --- | --- | --- | --- |
| **Trajectories of prenatal anxiety symptoms** | **Total (N)** | **0-6 months** | | **7-12 months** | | **13-24 months** | | ***P* for interaction** |
|  |  | **N (%)** | **RR (95% CI)^1^** | **N (%)** | **RR (95% CI)^1^** | **N (%)** | **RR (95% CI)^1^** |  |
| **Communication** |  |  |  |  |  |  |  |  |
| Low-stable | 2,799 | 2,361 (48.01) | Ref. | 359 (45.91) | Ref. | 79 (40.93) | Ref. |  |
| Moderate-stable | 1,546 | 1,268 (25.78) | 1.00 (0.82, 1.21) | 220 (28.13) | 1.38 (0.76, 2.50) | 58 (30.05) | 1.09 (0.30, 3.92) | 0.593 |
| Moderate-considerably decreasing | 1,038 | 879 (17.87) | 1.05 (0.85, 1.31) | 128 (16.37) | 1.47 (0.75, 2.87) | 31 (16.06) | 1.64 (0.43, 6.22) | 0.555 |
| High-slightly decreasing | 510 | 410 (8.34) | 1.24 (0.95, 1.63) | 75 (9.59) | 2.08 (1.04, 4.18) | 25 (12.95) | 0.61 (0.08, 4.94) | 0.309 |
| **Gross motor** |  |  |  |  |  |  |  |  |
| Low-stable | 2,799 | 2,361 (48.01) | Ref. | 359 (45.91) | Ref. | 79 (40.93) | Ref. |  |
| Moderate-stable | 1,546 | 1,268 (25.78) | 1.23 (0.96, 1.58) | 220 (28.13) | 0.89 (0.53, 1.51) | 58 (30.05) | 1.37 (0.42, 4.50) | 0.540 |
| Moderate-considerably decreasing | 1,038 | 879 (17.87) | 0.82 (0.59, 1.14) | 128 (16.37) | 0.82 (0.43, 1.55) | 31 (16.06) | 1.09 (0.23, 5.16) | 0.939 |
| High-slightly decreasing | 510 | 410 (8.34) | 1.38 (0.96, 1.98) | 75 (9.59) | 0.91 (0.42, 1.97) | 25 (12.95) | NA | 0.334 |
| **Fine motor** |  |  |  |  |  |  |  |  |
| Low-stable | 2,799 | 2,361 (48.01) | Ref. | 359 (45.91) | Ref. | 79 (40.93) | Ref. |  |
| Moderate-stable | 1,546 | 1,268 (25.78) | 1.28 (1.07, 1.54) | 220 (28.13) | 1.71 (0.61, 4.82) | 58 (30.05) | 2.03 (0.60, 6.79) | 0.667 |
| Moderate-considerably decreasing | 1,038 | 879 (17.87) | 1.08 (0.87, 1.34) | 128 (16.37) | 3.79 (1.49, 9.65) | 31 (16.06) | 3.94 (1.21, 12.80) | 0.005 |
| High-slightly decreasing | 510 | 410 (8.34) | 1.36 (1.04, 1.77) | 75 (9.59) | 5.89 (2.28, 15.23) | 25 (12.95) | 2.23 (0.53, 9.40) | 0.012 |
| **Problem solving** |  |  |  |  |  |  |  |  |
| Low-stable | 2,799 | 2,361 (48.01) | Ref. | 359 (45.91) | Ref. | 79 (40.93) | Ref. |  |
| Moderate-stable | 1,546 | 1,268 (25.78) | 1.31 (1.04, 1.65) | 220 (28.13) | 1.53 (0.63, 3.67) | 58 (30.05) | 0.59 (0.16, 2.19) | 0.467 |
| Moderate-considerably decreasing | 1,038 | 879 (17.87) | 0.87 (0.65, 1.18) | 128 (16.37) | 1.07 (0.34, 3.36) | 31 (16.06) | 1.16 (0.31, 4.27) | 0.876 |
| High-slightly decreasing | 510 | 410 (8.34) | 1.46 (1.05, 2.03) | 75 (9.59) | 0.95 (0.21, 4.28) | 25 (12.95) | 0.42 (0.05, 3.40) | 0.449 |
| **Personal-social** |  |  |  |  |  |  |  |  |
| Low-stable | 2,799 | 2,361 (48.01) | Ref. | 359 (45.91) | Ref. | 79 (40.93) | Ref. |  |
| Moderate-stable | 1,546 | 1,268 (25.78) | 1.22 (1.01, 1.47) | 220 (28.13) | 1.11 (0.58, 2.12) | 58 (30.05) | 0.96 (0.32, 2.81) | 0.879 |
| Moderate-considerably decreasing | 1,038 | 879 (17.87) | 0.99 (0.79, 1.25) | 128 (16.37) | 0.90 (0.39, 2.07) | 31 (16.06) | 1.14 (0.31, 4.10) | 0.955 |
| High-slightly decreasing | 510 | 410 (8.34) | 1.35 (1.03, 1.76) | 75 (9.59) | 1.31 (0.55, 3.11) | 25 (12.95) | 1.33 (0.37, 4.82) | 0.998 |
| Abbreviations: RR=Risk Ratio; CI= Confidence Interval; NA=Not available. | | | | | | | | |
| ^1^ Model adjusted for age at delivery, educational level, parity, mode of conception, pre-pregnancy body mass index, diabetic diseases, hypertensive diseases, and vaginal bleeding during early pregnancy of the mother, as well as sex and calendar year of birth of the child. | | | | | | | | |

| **Supplementary Table S11. Association between prenatal depressive symptoms across the three trimesters of pregnancy and risk of neurodevelopmental delay among children stratified by sex** | | | | | | |
| --- | --- | --- | --- | --- | --- | --- |
| **Prenatal depressive symptoms** | **Total (N)** | **RR (95% CI)^1^** | | | | |
|  |  | **Communication** | **Gross motor** | **Fine motor** | **Problem-solving** | **Personal-social** |
| **First trimester** |  |  |  |  |  |  |
| Male | 5,207 | 1.35 (1.14, 1.60) | 1.35 (1.09, 1.67) | 1.42 (1.20, 1.68) | 1.13 (0.91, 1.40) | 1.27 (1.07, 1.50) |
| Female | 4,521 | 1.02 (0.84, 1.24) | 1.06 (0.84, 1.32) | 1.20 (1.00, 1.45) | 0.88 (0.69, 1.12) | 1.07 (0.88, 1.29) |
| *P* for interaction |  | 0.031 | 0.116 | 0.199 | 0.120 | 0.178 |
| **Second trimester** |  |  |  |  |  |  |
| Male | 6,013 | 1.16 (0.94, 1.42) | 1.54 (1.19, 1.97) | 1.49 (1.23, 1.81) | 1.41 (1.09, 1.79) | 1.15 (0.92, 1.41) |
| Female | 5,145 | 1.15 (0.90, 1.45) | 1.20 (0.88, 1.61) | 1.29 (1.02, 1.62) | 1.16 (0.84, 1.55) | 1.26 (0.98, 1.59) |
| *P* for interaction |  | 0.939 | 0.214 | 0.349 | 0.328 | 0.570 |
| **Third trimester** |  |  |  |  |  |  |
| Male | 6,477 | 1.10 (0.89, 1.34) | 1.39 (1.08, 1.77) | 1.40 (1.15, 1.69) | 1.31 (1.02, 1.66) | 1.24 (1.01, 1.51) |
| Female | 5,653 | 1.30 (1.04, 1.61) | 1.03 (0.76, 1.37) | 1.41 (1.14, 1.74) | 1.32 (1.00, 1.71) | 1.50 (1.21, 1.85) |
| *P* for interaction |  | 0.259 | 0.128 | 0.959 | 0.971 | 0.200 |
| Abbreviations: RR=Risk Ratio; CI= Confidence Interval. | | | | | | |
| ^1^ Model adjusted for age at delivery, educational level, parity, mode of conception, pre-pregnancy body mass index, diabetic diseases, hypertensive diseases, and vaginal bleeding during early pregnancy of the mother, as well as calendar year of birth of the child. | | | | | | |

| **Supplementary Table S12. Association between prenatal anxiety symptoms across the three trimesters of pregnancy and risk of neurodevelopmental delay among children stratified by sex** | | | | | | |
| --- | --- | --- | --- | --- | --- | --- |
| **Prenatal anxiety symptoms** | **Total (N)** | **RR (95% CI)^1^** | | | | |
|  |  | **Communication** | **Gross motor** | **Fine motor** | **Problem-solving** | **Personal-social** |
| **First trimester** |  |  |  |  |  |  |
| Male | 5,207 | 1.16 (0.94, 1.43) | 1.43 (1.11, 1.82) | 1.39 (1.14, 1.70) | 1.24 (0.96, 1.60) | 1.09 (0.88, 1.34) |
| Female | 4,521 | 1.12 (0.88, 1.41) | 1.16 (0.87, 1.53) | 1.08 (0.85, 1.36) | 1.24 (0.93, 1.64) | 1.01 (0.78, 1.29) |
| *P* for interaction |  | 0.822 | 0.274 | 0.106 | 0.992 | 0.654 |
| **Second trimester** |  |  |  |  |  |  |
| Male | 6,013 | 1.11 (0.86, 1.42) | 1.58 (1.17, 2.08) | 1.55 (1.23, 1.92) | 1.21 (0.88, 1.63) | 1.37 (1.07, 1.73) |
| Female | 5,145 | 1.08 (0.79, 1.44) | 1.12 (0.76, 1.60) | 1.25 (0.94, 1.64) | 1.10 (0.74, 1.57) | 1.29 (0.96, 1.70) |
| *P* for interaction |  | 0.872 | 0.154 | 0.249 | 0.694 | 0.734 |
| **Third trimester** |  |  |  |  |  |  |
| Male | 6,477 | 0.97 (0.75, 1.23) | 1.45 (1.09, 1.91) | 1.39 (1.11, 1.73) | 1.57 (1.19, 2.02) | 1.47 (1.18, 1.82) |
| Female | 5,653 | 1.23 (0.94, 1.59) | 1.13 (0.80, 1.57) | 1.20 (0.92, 1.54) | 1.10 (0.77, 1.53) | 1.25 (0.96, 1.62) |
| *P* for interaction |  | 0.182 | 0.266 | 0.386 | 0.108 | 0.357 |
| Abbreviations: RR=Risk Ratio; CI= Confidence Interval. | | | | | | |
| ^1^Model adjusted for age at delivery, educational level, parity, mode of conception, pre-pregnancy body mass index, diabetic diseases, hypertensive diseases, and vaginal bleeding during early pregnancy of the mother, as well as calendar year of birth of the child. | | | | | | |

| **Supplementary Table S13. Associations between trajectories of prenatal depressive symptoms and child's neurodevelopmental delay during 0-24 months stratified by sex** | | | | | | |
| --- | --- | --- | --- | --- | --- | --- |
| **Trajectories of prenatal depressive symptoms** | **Total (N)** | **Male** | | **Female** | | ***P* for interaction** |
|  |  | **N (%)** | **RR (95% CI)^1^** | **N (%)** | **RR (95% CI)^1^** |  |
| **Communication** |  |  |  |  |  |  |
| Low-slightly decreasing | 2,177 | 1,150 (52.82) | Ref | 1,027 (47.18) | Ref |  |
| Moderate-slightly decreasing | 2,603 | 1,389 (53.36) | 1.19 (0.93, 1.52) | 1,214 (46.64) | 1.09 (0.84, 1.42) | 0.648 |
| Moderate-considerably decreasing | 452 | 227 (50.22) | 1.11 (0.69, 1.69) | 225 (49.78) | 1.07 (0.66, 1.66) | 0.914 |
| High-slightly decreasing | 661 | 351 (53.10) | 1.29 (0.90, 1.83) | 310 (46.90) | 1.48 (1.02, 2.10) | 0.612 |
| **Gross motor** |  |  |  |  |  |  |
| Low-slightly decreasing | 2,177 | 1,150 (52.82) | Ref | 1,027 (47.18) | Ref |  |
| Moderate-slightly decreasing | 2,603 | 1,389 (53.36) | 1.14 (0.84, 1.56) | 1,214 (46.64) | 1.10 (0.80, 1.52) | 0.889 |
| Moderate-considerably decreasing | 452 | 227 (50.22) | 1.35 (0.78, 2.22) | 225 (49.78) | 1.13 (0.63, 1.91) | 0.649 |
| High-slightly decreasing | 661 | 351 (53.10) | 1.58 (1.03, 2.38) | 310 (46.90) | 0.72 (0.39, 1.24) | 0.028 |
| **Fine motor** |  |  |  |  |  |  |
| Low-slightly decreasing | 2,177 | 1,150 (52.82) | Ref | 1,027 (47.18) | Ref |  |
| Moderate-slightly decreasing | 2,603 | 1,389 (53.36) | 1.31 (1.02, 1.69) | 1,214 (46.64) | 1.24 (0.95, 1.62) | 0.769 |
| Moderate-considerably decreasing | 452 | 227 (50.22) | 1.06 (0.64, 1.67) | 225 (49.78) | 0.86 (0.49, 1.40) | 0.555 |
| High-slightly decreasing | 661 | 351 (53.10) | 2.09 (1.51, 2.86) | 310 (46.90) | 1.75 (1.22, 2.47) | 0.463 |
| **Problem solving** |  |  |  |  |  |  |
| Low-slightly decreasing | 2,177 | 1,150 (52.82) | Ref | 1,027 (47.18) | Ref |  |
| Moderate-slightly decreasing | 2,603 | 1,389 (53.36) | 1.14 (0.85, 1.55) | 1,214 (46.64) | 1.08 (0.78, 1.49) | 0.781 |
| Moderate-considerably decreasing | 452 | 227 (50.22) | 0.63 (0.29, 1.19) | 225 (49.78) | 0.72 (0.35, 1.34) | 0.779 |
| High-slightly decreasing | 661 | 351 (53.10) | 1.62 (1.07, 2.41) | 310 (46.90) | 1.32 (0.82, 2.07) | 0.512 |
| **Personal-social** |  |  |  |  |  |  |
| Low-slightly decreasing | 2,177 | 1,150 (52.82) | Ref | 1,027 (47.18) | Ref |  |
| Moderate-slightly decreasing | 2,603 | 1,389 (53.36) | 1.31 (1.03, 1.68) | 1,214 (46.64) | 1.17 (0.90, 1.54) | 0.535 |
| Moderate-considerably decreasing | 452 | 227 (50.22) | 1.32 (0.85, 1.97) | 225 (49.78) | 0.80 (0.46, 1.33) | 0.151 |
| High-slightly decreasing | 661 | 351 (53.10) | 1.31 (0.91, 1.86) | 310 (46.90) | 1.38 (0.93, 2.01) | 0.836 |
| Abbreviations: RR=Risk Ratio; CI= Confidence Interval. | | | | | | |
| ^1^ Model adjusted for age at delivery, educational level, parity, mode of conception, pre-pregnancy body mass index, diabetic diseases, hypertensive diseases, and vaginal bleeding during early pregnancy of the mother, as well as calendar year of birth of the child. | | | | | | |
| **Supplementary Table S14. Associations between trajectories of prenatal anxiety symptoms and child's neurodevelopmental delay during 0-24 months stratified by sex** | | | | | | |
| **Trajectories of prenatal anxiety symptoms** | **Total (N)** | **Male** | | **Female** | | ***P* for interaction** |
|  |  | **N (%)** | **RR (95% CI)^1^** | **N (%)** | **RR (95% CI)^1^** |  |
| **Communication** |  |  |  |  |  |  |
| Low-stable | 2,799 | 1,481 (52.91) | Ref | 1,318 (47.09) | Ref |  |
| Moderate-stable | 1,546 | 814 (52.65) | 1.13 (0.86, 1.48) | 732 (47.35) | 0.93 (0.69, 1.23) | 0.310 |
| Moderate-considerably decreasing | 1,038 | 532 (51.25) | 1.13 (0.83, 1.53) | 506 (48.75) | 1.06 (0.77, 1.45) | 0.776 |
| High-slightly decreasing | 510 | 290 (56.86) | 1.47 (1.02, 2.06) | 220 (43.14) | 1.09 (0.70, 1.63) | 0.278 |
| **Gross motor** |  |  |  |  |  |  |
| Low-stable | 2,799 | 1,481 (52.91) | Ref | 1,318 (47.09) | Ref |  |
| Moderate-stable | 1,546 | 814 (52.65) | 1.31 (0.95, 1.80) | 732 (47.35) | 1.03 (0.73, 1.44) | 0.304 |
| Moderate-considerably decreasing | 1,038 | 532 (51.25) | 0.92 (0.60, 1.37) | 506 (48.75) | 0.73 (0.46, 1.11) | 0.451 |
| High-slightly decreasing | 510 | 290 (56.86) | 1.63 (1.06, 2.45) | 220 (43.14) | 0.77 (0.40, 1.35) | 0.044 |
| **Fine motor** |  |  |  |  |  |  |
| Low-stable | 2,799 | 1,481 (52.91) | Ref | 1,318 (47.09) | Ref |  |
| Moderate-stable | 1,546 | 814 (52.65) | 1.29 (0.98, 1.68) | 732 (47.35) | 1.32 (1.00, 1.72) | 0.906 |
| Moderate-considerably decreasing | 1,038 | 532 (51.25) | 1.51 (1.13, 2.01) | 506 (48.75) | 0.89 (0.62, 1.24) | 0.019 |
| High-slightly decreasing | 510 | 290 (56.86) | 1.71 (1.20, 2.38) | 220 (43.14) | 1.25 (0.81, 1.87) | 0.260 |
| **Problem solving** |  |  |  |  |  |  |
| Low-stable | 2,799 | 1,481 (52.91) | Ref | 1,318 (47.09) | Ref |  |
| Moderate-stable | 1,546 | 814 (52.65) | 1.21 (0.88, 1.67) | 732 (47.35) | 1.37 (0.98, 1.91) | 0.605 |
| Moderate-considerably decreasing | 1,038 | 532 (51.25) | 0.97 (0.64, 1.41) | 506 (48.75) | 0.82 (0.51, 1.26) | 0.581 |
| High-slightly decreasing | 510 | 290 (56.86) | 1.41 (0.89, 2.14) | 220 (43.14) | 1.28 (0.73, 2.11) | 0.787 |
| **Personal-social** |  |  |  |  |  |  |
| Low-stable | 2,799 | 1,481 (52.91) | Ref | 1,318 (47.09) | Ref |  |
| Moderate-stable | 1,546 | 814 (52.65) | 1.13 (0.87, 1.46) | 732 (47.35) | 1.29 (0.97, 1.70) | 0.500 |
| Moderate-considerably decreasing | 1,038 | 532 (51.25) | 1.07 (0.78, 1.43) | 506 (48.75) | 0.90 (0.62, 1.28) | 0.477 |
| High-slightly decreasing | 510 | 290 (56.86) | 1.34 (0.93, 1.88) | 220 (43.14) | 1.33 (0.86, 1.99) | 0.977 |
| Abbreviations: RR=Risk Ratio; CI= Confidence Interval. | | | | | | |
| ^1^Model adjusted for age at delivery, educational level, parity, mode of conception, pre-pregnancy body mass index, diabetic diseases, hypertensive diseases, and vaginal bleeding during early pregnancy of the mother, as well as calendar year of birth of the child. | | | | | | |

| **Supplementary Table S15. Characteristics of the children with and without at least one maternal psychological assessment during pregnancy** | | |  |
| --- | --- | --- | --- |
| **Characteristics** | **At least one maternal psychological assessment during pregnancy** | |  |
|  | **Yes (N=46,820)**  **N (%)** | **No (N=22,039)**  **N (%)** |  |
| **Mothers** |  |  |  |
| **Age at delivery (years)** |  |  |  |
| As continuous variable (mean [SD]) | 30.59 (3.93) | 30.39 (4.27) |  |
| <25 | 3,906 (8.34) | 2,547 (11.56) |  |
| 25-29 | 15,230 (32.53) | 7,045 (31.97) |  |
| 30-34 | 20,414 (43.60) | 8,797 (39.92) |  |
| ≥35 | 7,270 (15.53) | 3,650 (16.56) |  |
| **Educational level** |  |  |  |
| Senior high school or below | 8,983 (19.19) | 5,404 (24.52) |  |
| Undergraduate | 32,557 (69.54) | 12,849 (58.30) |  |
| Graduate | 2,979 (6.36) | 1,082 (4.91) |  |
| Missing | 2,301 (4.91) | 2,704 (12.27) |  |
| **Parity** |  |  |  |
| 1 | 26,342 (56.26) | 10,708 (48.59) |  |
| 2 | 17,209 (36.76) | 9,260 (42.02) |  |
| ≥3 | 3,268 (6.98) | 2,065 (9.37) |  |
| Missing | 1 (0.00) | 6 (0.03) |  |
| **Mode of conception** |  |  |  |
| Naturally conceived | 42,931 (91.69) | 19,198 (87.11) |  |
| ART | 2,010 (4.29) | 834 (3.78) |  |
| Missing | 1,879 (4.01) | 2,007 (9.11) |  |
| **Pre-pregnancy BMI** |  |  |  |
| As continuous variable (mean [SD]) | 21.10 (2.88) | 21.10 (2.99) |  |
| <18.5 | 7,433 (15.88) | 3,472 (15.75) |  |
| 18.5-23.9 | 30,914 (66.03) | 13,482 (61.17) |  |
| 24.0-27.9 | 5,378 (11.49) | 2,407 (10.92) |  |
| ≥28.0 | 1,110 (2.37) | 531 (2.41) |  |
| Missing | 1,985 (4.24) | 2,147 (9.74) |  |
| **Diabetic diseases** |  |  |  |
| No | 36,677 (78.34) | 17,650 (80.09) |  |
| Yes | 10,143 (21.66) | 4,389 (19.91) |  |
| **Hypertensive diseases** |  |  |  |
| No | 42,984 (91.81) | 20,517 (93.09) |  |
| Yes | 3,836 (8.19) | 1,522 (6.91) |  |
| **Vaginal bleeding during early pregnancy** |  |  |  |
| No | 31,819 (67.96) | 11,395 (51.70) |  |
| Yes | 6,978 (14.90) | 2,716 (12.32) |  |
| Missing | 8,023 (17.14) | 7,928 (35.97) |  |
| **Children** |  |  |  |
| **Sex** |  |  |  |
| Male | 24,803 (52.98) | 11,791 (53.50) |  |
| Female | 22,017 (47.02) | 10,248 (46.50) |  |
| **Gestational age at birth (weeks)** |  |  |  |
| As continuous variable (mean [SD]) | 38.80 (1.43) | 38.64 (1.79) |  |
| ≤33 | 412 (0.88) | 462 (2.10) |  |
| 34-36 | 1,742 (3.72) | 983 (4.46) |  |
| 37-38 | 13,701 (29.26) | 6,519 (29.58) |  |
| 39-40 | 28,033 (59.87) | 12,567 (57.02) |  |
| ≥41 | 2,932 (6.26) | 1,508 (6.84) |  |
| **Birth weight (grams)** |  |  |  |
| As continuous variable (mean [SD]) | 3,222.53 (429.00) | 3,204.91 (485.86) | |
| <2,500 | 1,779 (3.80) | 1,231 (5.59) |  |
| 2,500-2,999 | 10,312 (22.02) | 4,665 (21.17) |  |
| 3,000-3,499 | 22,598 (48.27) | 10,321 (46.83) |  |
| 3,500-3,999 | 10,476 (22.38) | 4,969 (22.55) |  |
| ≥4,000 | 1,655 (3.53) | 853 (3.87) |  |
| **Calendar year of birth** |  |  |  |
| 2020 | 4,265 (9.11) | 13,464 (61.09) |  |
| 2021 | 12,987 (27.74) | 4,599 (20.87) |  |
| 2022 | 15,024 (32.09) | 2,215 (10.05) |  |
| 2023 | 14,544 (31.06) | 1,761 (7.99) |  |
| Abbreviations: SD=Standard Deviation; ART=Assisted Reproductive Technology; BMI=Body Mass Index. | | |  |
|  | | |  |

| **Supplementary Table S16. Characteristics of the children with and without ASQ-3 assessment** | | |
| --- | --- | --- |
| **Characteristics** | **With ASQ-3**  **(N=16,229) N (%)** | **Without ASQ-3 (N=30,591) N (%)** |
| **Mothers** |  |  |
| **Age at delivery (years)** |  |  |
| As continuous variable (mean [SD]) | 30.35 (3.76) | 30.72 (4.01) |
| <25 | 1,297 (7.99) | 2,609 (8.53) |
| 25-29 | 5,792 (35.69) | 9,438 (30.85) |
| 30-34 | 6,958 (42.87) | 13,456 (43.99) |
| ≥35 | 2,182 (13.45) | 5,088 (16.63) |
| **Educational level** |  |  |
| Senior high school or below | 2,464 (15.18) | 6,519 (21.31) |
| Undergraduate | 12,267 (75.59) | 20,290 (66.33) |
| Graduate | 1,262 (7.78) | 1,717 (5.61) |
| Missing | 236 (1.45) | 2,065 (6.75) |
| **Parity** |  |  |
| 1 | 11,302 (69.64) | 15,040 (49.16) |
| 2 | 4,347 (26.79) | 12,862 (42.05) |
| ≥3 | 580 (3.57) | 2,688 (8.79) |
| Missing | 0 (0.00) | 1 (0.00) |
| **Mode of conception** |  |  |
| Naturally conceived | 15,189 (93.59) | 27,742 (90.69) |
| ART | 810 (4.99) | 1,200 (3.92) |
| Missing | 230 (1.42) | 1,649 (5.39) |
| **Pre-pregnancy BMI** |  |  |
| As continuous variable (mean [SD]) | 20.93 (2.78) | 21.20 (2.93) |
| <18.5 | 2,813 (17.33) | 4,620 (15.10) |
| 18.5-23.9 | 11,131 (68.59) | 19,783 (64.67) |
| 24.0-27.9 | 1,734 (10.68) | 3,644 (11.91) |
| ≥28.0 | 312 (1.92) | 798 (2.61) |
| Missing | 239 (1.47) | 1,746 (5.71) |
| **Diabetic diseases** |  |  |
| No | 12,685 (78.16) | 23,992 (78.43) |
| Yes | 3,544 (21.84) | 6,599 (21.57) |
| **Hypertensive diseases** |  |  |
| No | 14,900 (91.81) | 28,084 (91.80) |
| Yes | 1,329 (8.19) | 2,507 (8.20) |
| **Vaginal bleeding during early pregnancy** |  |  |
| No | 11,731 (72.28) | 20,088 (65.67) |
| Yes | 2,641 (16.27) | 4,337 (14.18) |
| Missing | 1,857 (11.44) | 6,166 (20.16) |
| **Children** |  |  |
| **Sex** |  |  |
| Male | 8,731 (53.80) | 16,072 (52.54) |
| Female | 7,498 (46.20) | 14,519 (47.46) |
| **Gestational age at birth (weeks)** |  |  |
| As continuous variable (mean [SD]) | 38.85 (1.33) | 38.77 (1.48) |
| ≤33 | 89 (0.55) | 323 (1.06) |
| 34-36 | 615 (3.79) | 1,127 (3.68) |
| 37-38 | 4,660 (28.71) | 9,041 (29.55) |
| 39-40 | 9,819 (60.50) | 18,214 (59.54) |
| ≥41 | 1,046 (6.45) | 1,886 (6.17) |
| **Birth weight (grams)** |  |  |
| As continuous variable (mean [SD]) | 3,217.76 (411.54) | 3,225.06 (437.97) |
| <2,500 | 577 (3.56) | 1,202 (3.93) |
| 2,500-2,999 | 3,684 (22.70) | 6,628 (21.67) |
| 3,000-3,499 | 7,907 (48.72) | 14,691 (48.02) |
| 3,500-3,999 | 3,535 (21.78) | 6,941 (22.69) |
| ≥4,000 | 526 (3.24) | 1,129 (3.69) |
| **Calendar year of birth** |  |  |
| 2020 | 1,631 (10.05) | 2,634 (8.61) |
| 2021 | 4,990 (30.75) | 7,997 (26.14) |
| 2022 | 5,487 (33.81) | 9,537 (31.18) |
| 2023 | 4,121 (25.39) | 10,423 (34.07) |
| Abbreviations: ASQ-3=Ages and Stages Questionnaire-Third Edition; SD=Standard Deviation; ART=Assisted Reproductive Technology; BMI=Body Mass Index. | | |

| **Supplementary Table S17. Characteristics of the children with and without complete three maternal psychological assessments during pregnancy** | | |
| --- | --- | --- |
| **Characteristic** | **Complete three maternal psychological assessments during pregnancy** | |
|  | **Yes (N=5,893)**  **N (%)** | **No (N=10,336)**  **N (%)** |
| **Mothers** |  |  |
| **Age at delivery (years)** |  |  |
| As continuous variable (mean [SD]) | 30.49 (3.62) | 30.27 (3.84) |
| <25 | 393 (6.67) | 904 (8.75) |
| 25-29 | 2,064 (35.02) | 3,728 (36.07) |
| 30-34 | 2,645 (44.88) | 4,313 (41.73) |
| ≥35 | 791 (13.42) | 1,391 (13.46) |
| **Educational level** |  |  |
| Senior high school or below | 716 (12.15) | 1,748 (16.91) |
| Undergraduate | 4,533 (76.92) | 7,734 (74.83) |
| Graduate | 485 (8.23) | 777 (7.52) |
| Missing | 159 (2.70) | 77 (0.74) |
| **Parity** |  |  |
| 1 | 4,241 (71.97) | 7,061 (68.31) |
| 2 | 1,457 (24.72) | 2,890 (27.96) |
| ≥3 | 195 (3.31) | 385 (3.72) |
| **Mode of conception** |  |  |
| Naturally conceived | 5,461 (92.67) | 9,728 (94.12) |
| ART | 275 (4.67) | 535 (5.18) |
| Missing | 157 (2.66) | 73 (0.71) |
| **Pre-pregnancy BMI** |  |  |
| As continuous variable (mean [SD]) | 21.01 (2.81) | 20.89 (2.76) |
| <18.5 | 953 (16.17) | 1,860 (18.00) |
| 18.5-23.9 | 4,024 (68.28) | 7,107 (68.76) |
| 24.0-27.9 | 619 (10.50) | 1,115 (10.79) |
| ≥28.0 | 135 (2.29) | 177 (1.71) |
| Missing | 162 (2.75) | 77 (0.74) |
| **Diabetic diseases** |  |  |
| No | 4,668 (79.21) | 8,017 (77.56) |
| Yes | 1,225 (20.79) | 2,319 (22.44) |
| **Hypertensive diseases** |  |  |
| No | 5,351 (90.80) | 9,549 (92.39) |
| Yes | 542 (9.20) | 787 (7.61) |
| **Vaginal bleeding during early pregnancy** |  |  |
| No | 4,388 (74.46) | 7,343 (71.04) |
| Yes | 944 (16.02) | 1,697 (16.42) |
| Missing | 561 (9.52) | 1,296 (12.54) |
| **Children** |  |  |
| **Sex** |  |  |
| Male | 3,117 (52.89) | 5,614 (54.32) |
| Female | 2,776 (47.11) | 4,722 (45.68) |
| **Gestational age at birth (weeks)** |  |  |
| As continuous variable (mean [SD]) | 38.90 (1.21) | 38.81 (1.40) |
| ≤33 | 10 (0.17) | 79 (0.76) |
| 34-36 | 167 (2.83) | 448 (4.33) |
| 37-38 | 1,747 (29.65) | 2,913 (28.18) |
| 39-40 | 3,597 (61.04) | 6,222 (60.20) |
| ≥41 | 372 (6.31) | 674 (6.52) |
| **Birth weight (grams)** |  |  |
| As continuous variable (mean [SD]) | 3,220.98 (386.75) | 3,215.93 (425.04) |
| <2,500 | 165 (2.80) | 412 (3.99) |
| 2,500-2,999 | 1,358 (23.04) | 2,326 (22.50) |
| 3,000-3,499 | 2,910 (49.38) | 4,997 (48.35) |
| 3,500-3,999 | 1,291 (21.91) | 2,244 (21.71) |
| ≥4,000 | 169 (2.87) | 357 (3.45) |
| **Calendar year of birth** |  |  |
| 2020 | 15 (0.25) | 1,616 (15.63) |
| 2021 | 271 (4.60) | 4,719 (45.66) |
| 2022 | 2,450 (41.57) | 3,037 (29.38) |
| 2023 | 3,157 (53.57) | 964 (9.33) |
| **Prenatal psychological status** |  |  |
| **Depressive symptoms** |  |  |
| **First trimester** |  |  |
| As continuous variable (median [IQR]) | 3 (2, 6) | 4 (2, 7) |
| No | 3,760 (63.80) | 2,149 (56.04) |
| Yes | 2,133 (36.20) | 1,686 (43.96) |
| **Second trimester** |  |  |
| As continuous variable (median [IQR]) | 1 (0, 3) | 1 (0, 4) |
| No | 5,225 (88.66) | 4,281 (81.31) |
| Yes | 668 (11.34) | 9,84 (18.69) |
| **Third trimester** |  |  |
| As continuous variable (median [IQR]) | 1 (0, 3) | 2 (0, 4) |
| No | 5,146 (87.32) | 5,036 (80.74) |
| Yes | 747 (12.68) | 1,201 (19.26) |
| **Anxiety symptoms** |  |  |
| **First trimester** |  |  |
| As continuous variable (median [IQR]) | 1 (0, 3) | 1 (0, 4) |
| No | 4,913 (83.37) | 3,073 (80.13) |
| Yes | 980 (16.63) | 762 (19.87) |
| **Second trimester** |  |  |
| As continuous variable (median [IQR]) | 0 (0, 1) | 0 (0, 2) |
| No | 5,470 (92.82) | 4,632 (87.98) |
| Yes | 423 (7.18) | 633 (12.02) |
| **Third trimester** |  |  |
| As continuous variable (median [IQR]) | 0 (0, 2) | 1 (0, 3) |
| No | 5,431 (92.16) | 5,385 (86.34) |
| Yes | 462 (7.84) | 852 (13.66) |
| **ASQ-3 domains** |  |  |
| **Communication** |  |  |
| Normal | 5,211 (88.43) | 9,227 (89.27) |
| Delayed | 682 (11.57) | 1,109 (10.73) |
| **Gross motor** |  |  |
| Normal | 5,443 (92.36) | 9,659 (93.45) |
| Delayed | 450 (7.64) | 677 (6.55) |
| **Fine motor** |  |  |
| Normal | 5,193 (88.12) | 9,286 (89.84) |
| Delayed | 700 (11.88) | 1,050 (10.16) |
| **Problem-solving** |  |  |
| Normal | 5,435 (92.23) | 9,657 (93.43) |
| Delayed | 458 (7.77) | 679 (6.57) |
| **Personal-social** |  |  |
| Normal | 5,212 (88.44) | 9,293 (89.91) |
| Delayed | 681 (11.56) | 1,043 (10.09) |
| Abbreviations: SD=Standard Deviation; IQR=Interquartile range; ART=Assisted Reproductive Technology; BMI=Body Mass Index. | | |

| **Supplementary Table S18. Distribution of gestational age by completeness of maternal psychological assessment during each trimester of pregnancy** | | | |  |
| --- | --- | --- | --- | --- |
| **Maternal psychological assessments during pregnancy** | **Overall**  **(N [%])** | **Preterm births (<37 gestational weeks) (N [%]^1^)** | **Full-term births (≥37 gestational weeks) (N [%]^1^)** | |
| **With complete three assessments** | 5,893 (100.00) | 177 (3.00) | 5,716 (97.00) | |
| **Without complete three assessments** | 10,336 (100.00) | 527 (5.10) | 9,809 (94.90) | |
| Assessment during the first trimester |  |  |  | |
| Yes | 3,835 (37.10) | 264 (6.88) | 3,571 (93.12) | |
| No | 6,501 (62.90) | 263 (4.05) | 6,238 (95.95) | |
| Assessment during the second trimester |  |  |  | |
| Yes | 5,265 (50.94) | 311 (5.91) | 4,954 (94.09) | |
| No | 5,071 (49.06) | 216 (4.26) | 4,855 (95.74) | |
| Assessment during the third trimester |  |  |  | |
| Yes | 6,237 (60.34) | 180 (2.89) | 6,057 (97.11) | |
| No | 4,099 (39.66) | 347 (8.47) | 3,752 (91.53) | |
| ^1^ row percentage. | | | | |
